# Platelets are critical for survival and tissue integrity during murine pulmonary *Aspergillus fumigatus* infection

**DOI:** 10.1371/journal.ppat.1008544

**Published:** 2020-05-14

**Authors:** Benjamin Y. Tischler, Nicholas L. Tosini, Robert A. Cramer, Tobias M. Hohl

**Affiliations:** 1 Louis V. Gerstner, Jr. Graduate School of Biomedical Sciences, Memorial Sloan Kettering Cancer Center, New York, New York, United States of America; 2 Immunology Program, Sloan Kettering Institute, Memorial Sloan Kettering Cancer Center, New York, New York, United States of America; 3 Department of Microbiology and Immunology, Geisel School of Medicine at Dartmouth, Hanover, New Hampshire, United States of America; 4 Department of Medicine, Memorial Sloan Kettering Cancer Center, New York, New York, United States of America; University of Cincinnati College of Medicine, UNITED STATES

## Abstract

Beyond their canonical roles in hemostasis and thrombosis, platelets function in the innate immune response by interacting directly with pathogens and by regulating the recruitment and activation of immune effector cells. Thrombocytopenia often coincides with neutropenia in patients with hematologic malignancies and in allogeneic hematopoietic cell transplant recipients, patient groups at high risk for invasive fungal infections. While neutropenia is well established as a major clinical risk factor for invasive fungal infections, the role of platelets in host defense against human fungal pathogens remains understudied. Here, we examined the role of platelets in murine *Aspergillus fumigatus* infection using two complementary approaches to induce thrombocytopenia without concurrent neutropenia. Thrombocytopenic mice were highly susceptible to *A*. *fumigatus* challenge and rapidly succumbed to infection. Although platelets regulated early conidial phagocytosis by neutrophils in a spleen tyrosine kinase (Syk)-dependent manner, platelet-regulated conidial phagocytosis was dispensable for host survival. Instead, our data indicated that platelets primarily function to maintain hemostasis and lung integrity in response to exposed fungal antigens, since thrombocytopenic mice exhibited severe hemorrhage into the airways in response to fungal challenge in the absence of overt angioinvasion. Challenge with swollen, heat-killed, conidia was lethal in thrombocytopenic hosts and could be reversed by platelet transfusion, consistent with the model that fungus-induced inflammation in platelet-depleted mice was sufficient to induce lethal hemorrhage. These data provide new insights into the role of platelets in the anti-*Aspergillus* host response and expand their role to host defense against filamentous molds.

## Author summary

*Aspergillus fumigatus* is a ubiquitous environmental mold that forms airborne spores, termed conidia. When inhaled by immune compromised individuals, *A*. *fumigatus* conidia can germinate into tissue-invasive hyphae and cause invasive aspergillosis, a major cause of infectious morbidity and mortality in patients with leukemia and in bone marrow transplant recipients. Although a low platelet count has been identified as a risk factor for clinical outcomes in patients with invasive aspergillosis, the precise role of platelets in the anti-fungal host response remains poorly understood. Here, we report an essential requirement for platelets in anti-*Aspergillus* host defence in a mouse model of fungal pneumonia. Although platelets play a role in activating the innate immune system after infection, they are critical for preventing lethal hemorrhage after *A*. *fumigatus* challenge. Our findings raise the question as to whether platelets can be used as a basis for therapeutic strategies in vulnerable patient populations.

## Introduction

*Aspergillus fumigatus*, an environmental mold and opportunistic pathogen, reproduces by releasing asexual spores (conidia). Humans inhale hundreds to thousands of conidia daily. In immune competent individuals, conidia are cleared by the cells of the innate immune system, including alveolar macrophages, inflammatory monocytes, and neutrophils. Quantitative defects in neutrophil count (i.e., neutropenia), or qualitative defects in neutrophil function (e.g., corticosteroid treatment or chronic granulomatous disease), are the primary risk factors for developing invasive aspergillosis (IA) [1]. IA develops in an estimated 200,000 patients worldwide annually [2] and has high fungus-attributable mortality rates, typically 20–25% in high-risk groups [3], despite receipt of contemporary antifungal drugs.

Beyond neutropenia, thrombocytopenia has been identified as a risk factor for clinical outcomes in patients with IA [4]. As thrombocytopenia is often concomitant with other cytopenias, the exact role platelets play in the human host response to IA remains unknown [5,6]. In addition to their canonical roles in hemostasis, platelets are increasingly recognized as important players in the immune response [7]. Platelets express pattern recognition receptors [8], signal through spleen tyrosine kinase (Syk) [9,10], generate reactive oxygen species [11], and release chemokines and microbicidal peptides [7]. In response to bacterial challenge, platelets perform both antimicrobial and tissue protective functions [12].

Though in vitro studies identified putative roles for platelets in anti-*Aspergillus* immune defense [13], including exerting direct antifungal activity [14–19] and augmenting the activity of leukocytes [14,20–22], the in vivo role of platelets in anti-*Aspergillus* host defense and their contribution to innate immune defense functions at the portal of infection remain undefined. In this study, we integrated the role of platelets into a model of the pulmonary anti-*Aspergillus* immune response. Using complementary platelet depletion strategies, we uncovered an essential, non-redundant role for platelets in the host response to *Aspergillus* challenge. Thrombocytopenic mice were highly susceptible to both highly virulent and less virulent strains of *A*. *fumigatus*. Mortality was not associated with significant increases in fungal germination nor dissemination to extrapulmonary sites. In the lung, platelet depletion did not impair neutrophil recruitment, but did impact their early phagocytic response in a megakaryocyte lineage Syk-dependent signaling manner. Thrombocytopenic mice exhibited severe hemorrhage into the airways and lung parenchyma in response to *Aspergillus* challenge. Inoculating mice with swollen, heat-killed, conidia induced inflammation in the absence of fungal growth, and thrombocytopenic mice were highly susceptible to inflammatory challenge, a finding that supports the model that murine mortality is driven by inflammation-induced hemorrhage. Importantly, the lethal challenge was rescued by transfusion of purified platelets into thrombocytopenic mice. Together, our data suggest that platelets play a major role in maintaining hemostasis and tissue integrity, while also enhancing neutrophil phagocytosis, during in vivo pulmonary *A*. *fumigatus* challenge.

## Materials and methods

### Chemicals and reagents

Unless otherwise noted, chemicals were purchased from Sigma-Aldrich or Fisher Scientific, cell culture reagents from Thermo Fisher Scientific, and microbiological culture media from BD Biosciences. Antibodies for flow cytometry were acquired from BD Biosciences, Thermo Fisher Scientific, and Tonbo.

### Mice

C57BL/6J (stock #00664), PF4-iCre (stock #008535), Syk^fl/fl^ (stock # 017309) and ROSA26iDTR (stock #007900) were purchased from the Jackson Laboratory (JAX). C57BL/6-Ly5.1 (CD45.1, stock #564) was purchased from Charles River Laboratories. Mice were allowed to adjust to housing in the MSKCC vivarium for one week. Age-matched, sex-matched, and/or littermate controls were used for experiments as appropriate. Animals were euthanized by either pentobarbital injection or CO_2_ per IACUC guidelines.

### Ethics statement

All animal studies were performed with approval from the MSKCC Institutional Animal Care and Use Committee under protocol 13-07-008 and were compliant with all applicable provisions established by the Animal Welfare Act and the Public Health Services (PHS) Policy on the Humane Care and Use of Laboratory Animals.

### Generation of bone marrow chimeras

Bone marrow (BM) chimeric mice were generated by reconstituting lethally irradiated (9 Gy) CD45.1 mice with BM cells from either PF4-iCre^+^; Syk^fl/fl^ mice or Syk^fl/fl^ mice by tail vein injection. Approximately 1-5x10^6^ cells were used for each injection. Irradiated mice were maintained on water supplemented with Baytril for 3 weeks post irradiation. Mice were allowed to rest for 6 weeks prior to further experimentation.

### Depletion and monitoring of platelets

To model thrombocytopenia via antibody-mediated depletion, C57BL/6J mice were injected i.p. with 100 μL of rabbit anti-mouse platelet serum (Accurate Chemical and Scientific Corporation, AIA31440) diluted 1:4, or an equivalent volume of diluted normal rabbit serum (Accurate Chemical and Scientific Corporation, JNZ000120).

To model thrombocytopenia by diphtheria toxin receptor-mediated megakaryocyte depletion, PF4-iCre mice were crossed to ROSA26iDTR (iDTR) mice (iDTR^Pf4^). On the first day of depletion, 400 ng of diphtheria toxin (DT) (List Biological Laboratories, Cat. No. 150) was injected i.p. into Cre-positive mice and Cre-negative littermates. On the second and fourth days of depletion, 200 ng of DT was injected i.p.

For both models, the degree of platelet depletion was monitored by anesthetizing mice and placing 2 μL of tail vain blood into FACS buffer containing an equivalent volume of fluorescent beads (Spherotech, Inc., ACFP-70-10) and FITC-conjugated anti-CD41 antibody and analyzing platelet numbers by flow cytometry.

### Preparation of conidia

CEA10, Af293, or Δ*pksP* (AF293.1 background) conidia were grown on glucose minimal media agar slants for approximately 7 days at 37 degrees Celsius. The Δ*pksP* strain was generated by replacing the *pksP* open reading frame with *A*. *niger pyrG* in the AF293.1 background and selecting for uracil/uridine prototrophs and white conidia. Single insertion of the *pyrG* marker and replacement of *pksP* were confirmed with PCR and Southern blot. Conidia were harvested from the slants using PBS with 0.025% Tween 20 and resuspended at the appropriate concentration for experimental use. For experiments involving FLARE conidia, DsRed Af293 conidia were grown on Sabouraud dextrose agar slants and labeled with Alexa Fluor 633 as previously described.

### Preparation of heat-killed conidia

To generate resting, heat-killed, conidia, harvested conidia were adjusted to the desired inoculum, and heat killed by boiling at 100 degrees Celsius for at least 30 minutes. Swollen, heat-killed, conidia were generated by overnight incubation at 37 degrees Celsius in RPMI medium supplemented with 0.5 μg/mL voriconazole before boiling and adjusting the concentration. Killing was confirmed by plating conidia.

### Fungal challenge of mice by intranasal inoculation

6-12-week-old mice, or in the case of BM chimeras, mice at least 6 weeks post-transplant, were anesthetized with isoflurane and 50 μL of conidial suspension was placed on the nares. Upon completion of inhalation, mice were returned to their cages and monitored for discomfort.

### Survival experiments

In survival experiments using antiserum, mice were injected 2 hours prior to infection and every 24 hours thereafter. In experiments involving DT, mice were infected 6 days after the initiation of depletion. Mice were monitored twice daily for the duration of experiment and euthanized when moribund. For some experiments, 1 x 10^8^ isolated platelets (see below) were infused in a volume of 200 μL through the tail vein 1 hour after infection.

### Analysis of neutrophil function

Mice were depleted as above and infected with FLARE conidia. After euthanasia, lungs were perfused using PBS through the right ventricle. Single cell lung suspensions were stained with fluorescent antibodies to Ly6G, Ly6C, CD11b, CD45, CD11c, MHCII, as well as a live-dead stain. Neutrophils were identified as live CD45^+^ CD11b^+^ Ly6G^+^ cells. Monocytes were identified as live CD45^+^ CD11b^+^ Ly6G^-^ Ly6C^+^ CD11c^-^ cells, and monocyte-derived dendritic cells were identified as live CD45^+^ CD11b^+^ Ly6G^-^ Ly6C^+^ CD11c^+^ MHCII^+^ cells. Data were acquired on a BD LSRII flow cytometer and analyzed on FlowJo (Treestar). Phagocytosis and fungicidal ability was assessed as previously described [23]. Cell counts were determined with a Z2 Coulter counter. To account for experimental variation, results were normalized to controls before pooling the data.

### Fungal burden

To assess fungal burden, mice were euthanized by CO_2_ asphyxiation and organs were homogenized in 2 mL PBS using a Power Gen 125 homogenizer (Fisher). Colony forming units (CFU) were determined by plating serial dilutions on Sabouraud dextrose agar plates.

### Histology

Mice were euthanized by pentobarbital (FatalPlus) and perfused with 8–10 mL of PBS through the right ventricle. The lungs were inflated using 4% paraformaldehyde (PFA) and placed in PFA for 72h at 4 degrees Celsius. The fixed tissues were then washed in deionized water and stored in 70% ethanol before processing by the MSKCC Molecular Cytology Core Facility to generate paraffin embedded sections, and stained with hematoxylin & eosin (H&E) or Grocott’s methenamine silver stain (GMS). Images were acquired by a Zeiss Mirax Midi slide scanner and analyzed using Pannoramic Viewer. Germination rate was determined by counting the number of germlings and hyphae, and dividing by the total number of fungal particles counted in ImageJ.

### Tissue damage and vascular permeability

Mice were depleted of platelets and challenged with fungi as indicated. Sixty minutes prior to euthanasia, mice were inoculated intranasally with 50 μL of 4 mg/mL FITC Dextran MW 400 (Sigma Aldrich, 46944). Mice were euthanized by FatalPlus. Blood was drawn into syringes containing 3.8% sodium citrate solution. Following blood collection, bronchoalveolar lavage fluid (BALF) was obtained by inserting a catheter into the trachea, followed by inflating and deflating the lungs using 3 mL PBS. The mouse was then perfused with PBS through the right ventricle and the lungs removed. Airway hemorrhage was determined by measuring absorbance of BALF at 410 nm, a peak characteristic of hemoglobin, using serial dilutions. Airway protein content was determined by BCG Albumin Assay according to manufacturer’s instructions (Sigma, MAK124). BALF LDH levels were determined by the CytoTox96 non-radioactive cytotoxicity assay kit according to manufacturer’s instructions (Promega, G1780). Vascular permeability was assessed by centrifuging peripheral blood at 7000 RPM for 10 minutes, placing 100 μL of plasma in an ELISA plate, and reading the MFI at 485 nm excitation and 528 nm emission [24]. To enable comparison across experiments, results from thrombocytopenic mice were normalized to control mice and pooled.

### Platelet isolation for Western Blot analysis and transfusion

To isolate platelets, blood was drawn from the vena cava into syringes containing 3.8% sodium citrate and pooled from multiple mice. The citrated blood was centrifuged at 100 x g for 10 minutes at room temperature (no brake for all centrifugations). The platelet-rich plasma was transferred to a new tube and mixed with an equivalent volume of HEP buffer (140mM NaCl, 2.7mM, 3.8 mM HEPES, 5 mM EGTA, pH 7.4) and 1 μM prostaglandin E1 (omitted for in vivo experiments). The platelet suspension was first centrifuged at 100 x g for 15 minutes to remove larger cells and then pelleted at 800 x g for 15 minutes. The platelets were washed twice in platelet wash buffer (10mM sodium citrate, 150 mM NaCl, 1mM EDTA, 1% (w/v) dextrose, pH 7.4) and resuspended in Tyrode’s buffer (130 mM NaCl, 12 mM NaHCO_3_, 2.9 mM KCl, 0.34 Na_2_HPO_4_, 1 mM MgCl_2_, 10 mM HEPES, pH 7.4) supplemented with 3 mg/mL BSA, 5 mM glucose, and 1 μM prostaglandin E1 (omitted from in vivo experiments) and enumerated by flow cytometry.

### Western Blot

Platelet lysate was prepared by incubating the platelet suspension with an equivalent volume of 2x lysis buffer (2% NP40, 30 mM HEPES, 150 mM NaCl, 2 mM EDTA, pH 7.4) and HALT protease and phosphatase inhibitor for 20 minutes on ice. The lysate was then clarified at 17,000 x g for 20 minutes. The supernatant was separated by SDS-PAGE and transferred to nitrocellulose membranes using Bolt MES reagents (Thermo Fisher Scientific). Membranes were probed for Syk (Cell Signaling Technologies, 2712) and actin (Cell Signaling Technologies, 3700). LiCor IRDye secondary antibodies were used to image membranes on a LiCor Odyssey Infrared Imager.

### Statistical analysis

All results are expressed as mean (± SEM) using pooled results from 1–3 independent experiments as noted in the text. For comparisons between two groups, a Mann-Whitney U test was used. For survival experiments, a Mantel-Cox test was used. All statistical analyses were performed with GraphPad Prism, version 7.0.

## Results

### Platelets are essential for survival following *A*. *fumigatus* challenge

**Fig 1:**
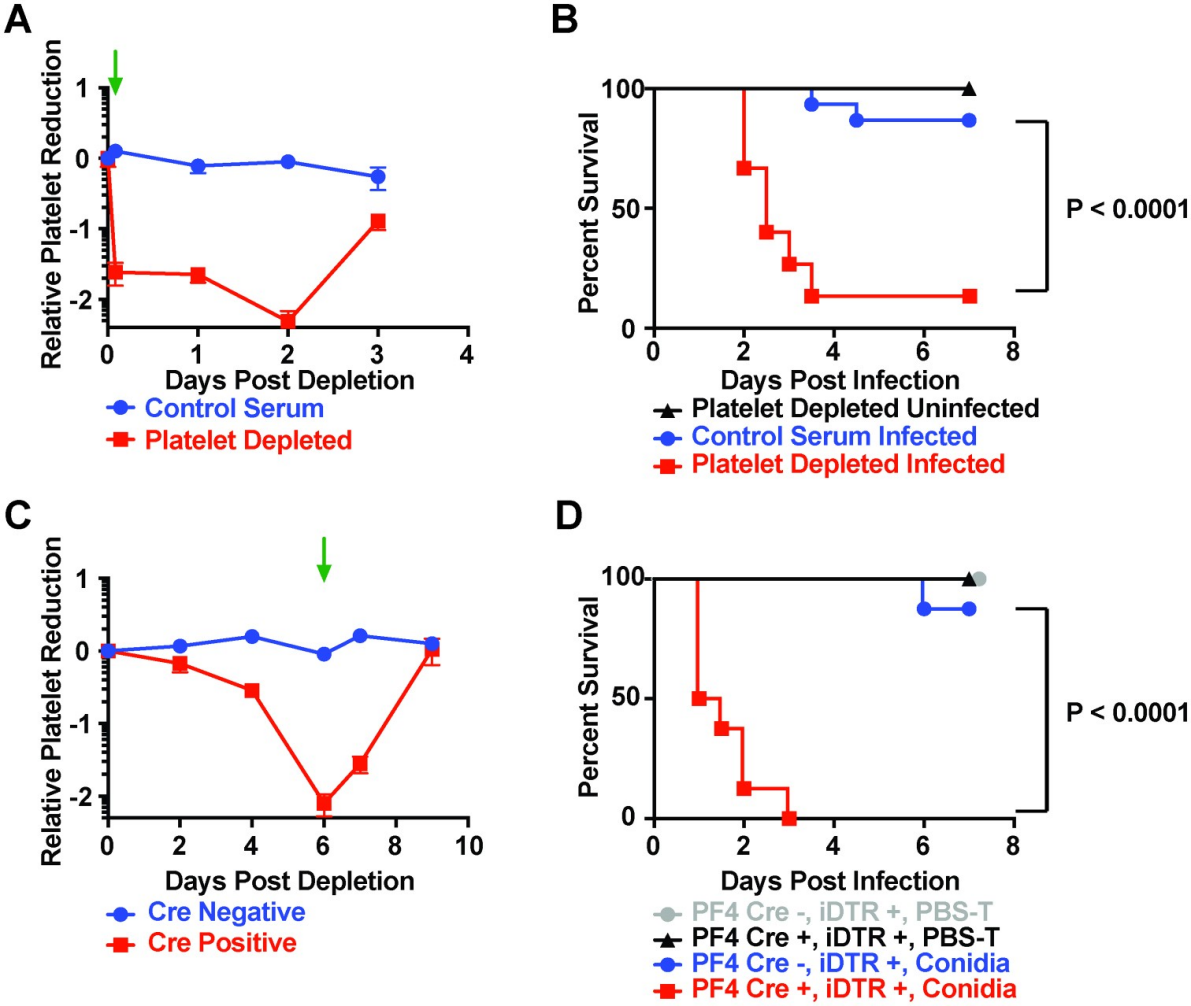
Platelets are essential for survival after *Aspergillus fumigatus* challenge. (A) Relative peripheral blood platelet levels (log_10_ scale) in C57BL/6J mice injected with platelet targeting serum (red) or normal rabbit serum (blue) on day 0, day +1, and day +2. (*n* = 4–5 mice). The green arrow indicates the timing of infection in B. (B) Kaplan-Meier survival curve of mice injected with normal rabbit serum (blue) or platelet targeting serum (red, black), that were infected with ~6x10^7^ *A*. *fumigatus* CEA10 conidia (blue, red) or left uninfected (black). Data are pooled from 2 experiments (*n* = 10–15 per group). (C) Relative peripheral blood platelet levels (log_10_ scale) of PF4-iCre-positive, iDTR positive, mice (red) or Cre-negative, iDTR positive, littermates (blue) injected with 400 ng diphtheria toxin (DT), and subsequent doses of DT on day 2 (200 ng), and day 4 (200 ng). (*n* = 4–6 mice per group). The green arrow indicates the timing of infection in D. (D) Kaplan-Meier survival curve of PF4-iCre positive or negative, iDTR positive mice, injected with DT and either infected with ~6x10^7^ *A*. *fumigatus* CEA10 conidia (blue, red) or left uninfected (grey, black). Uninfected mice were from 1 experiment (*n* = 4–6 mice per group), and infected mice were pooled from 2 experiments (*n* = 8 mice per group).

To induce thrombocytopenia in mice, we utilized two experimental strategies in parallel. In the first model, we induced thrombocytopenia by injecting mice with a platelet-targeting antiserum (AS model) on three consecutive days. In the AS model, platelet counts fell to < 3% of starting levels within 2 hours of the first injection (Figs 1A and S1A). In the AS model, the onset of platelet recovery began on day +3 (Figs 1A and S1A).

To assess the importance of platelets for survival in the AS model, we infected thrombocytopenic or control mice with the virulent *A*. *fumigatus* clinical isolate CEA10 2 hours after the initial antiserum administration. In the absence of thrombocytopenia, peripheral blood platelet levels declined in the first 24 hours of infection, though the decrease did not reach statistical significance, before recovering over the following 2 days (S1B Fig). Thirteen of fifteen thrombocytopenic mice succumbed to infection with a median survival time of 2.5 days (Fig 1B). Only two of fifteen platelet-sufficient mice died from infection (Fig 1B). Importantly, no uninfected thrombocytopenic mice died during the experiment, indicating that the platelet-depleting strategy did not account for murine mortality (Fig 1B). This phenotype was strain independent since infection with the less virulent clinical isolate Af293 yielded a similar result (S2 Fig). Four of five thrombocytopenic mice died in comparison to no mortality in the control group (S2 Fig).

In the second model of thrombocytopenia, we injected iDTR^Pf4^ mice or non-Cre littermate controls with 3 doses of diphtheria toxin, each two days apart (DT model). Six days after the initial DT dose, platelet levels were reduced to < 1% of normal levels in Cre-positive mice (Figs 1C and S1C). Mice remained thrombocytopenic for an additional 24 hours, before recovering normal platelet levels on day +9 (Figs 1C and S1C). To confirm that the infectious susceptibility in the AS model (Fig 1B) was not due to off-target effects, we challenged thrombocytopenic mice with CEA10 conidia in the DT model on day +6 after the first DT dose to target Pf4^+^ megakaryocytes. All thrombocytopenic mice infected with conidia died with a median survival time of one day, compared with only one of eight infected controls (Fig 1D). No uninfected controls died, indicating that mortality was not a result of the depletion strategy (Fig 1D). Regardless of the mechanism of platelet depletion, thrombocytopenic mice were highly susceptible to *A*. *fumigatus* challenge with 2 different clinical isolates.

**Fig 2:**
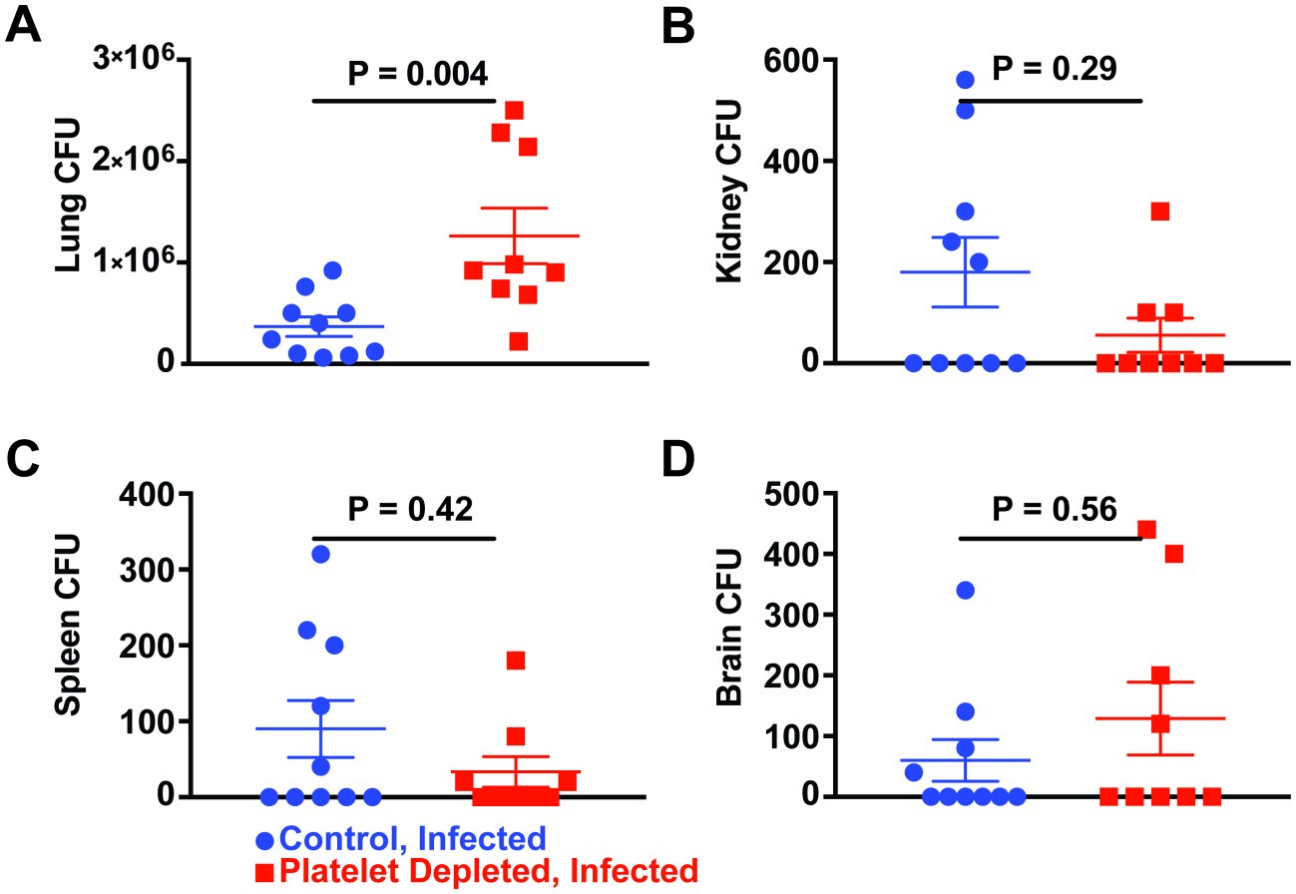
Platelet depletion and organ fungal burden. Colony forming units (CFU) in C57BL/6J mice treated with normal serum (blue) or platelet targeting antiserum (red) and infected with ~3-6x10^7^ *A*. *fumigatus* CEA10 conidia. CFU levels in the (A) lung, (B) kidney, (C) spleen, and (D) brain, 2 days after infection. Error bars are expressed with mean ± SEM. Data are pooled from 2 experiments (*n* = 9–10 mice per group).

To examine the impact of thrombocytopenia on fungal growth and dissemination in the AS model, we challenged thrombocytopenic or control mice with CEA10 conidia and quantified organ fungal colony-forming units (CFU) at 2 days post infection. We observed a higher lung fungal burden in thrombocytopenic mice (1,260,000 ± 270,000 CFU) compared with controls (370,000 ± 100,000 CFU) (Fig 2A). However, we did not observe notable dissemination to the kidney (Fig 2B), spleen (Fig 2C), or brain (Fig 2D). These data suggest that platelets contribute to controlling fungal growth in the lung, but that rapid mortality likely limits dissemination to extrapulmonary sites.

### Platelets regulate neutrophil function during *A*. *fumigatus* lung infection

**Fig 3:**
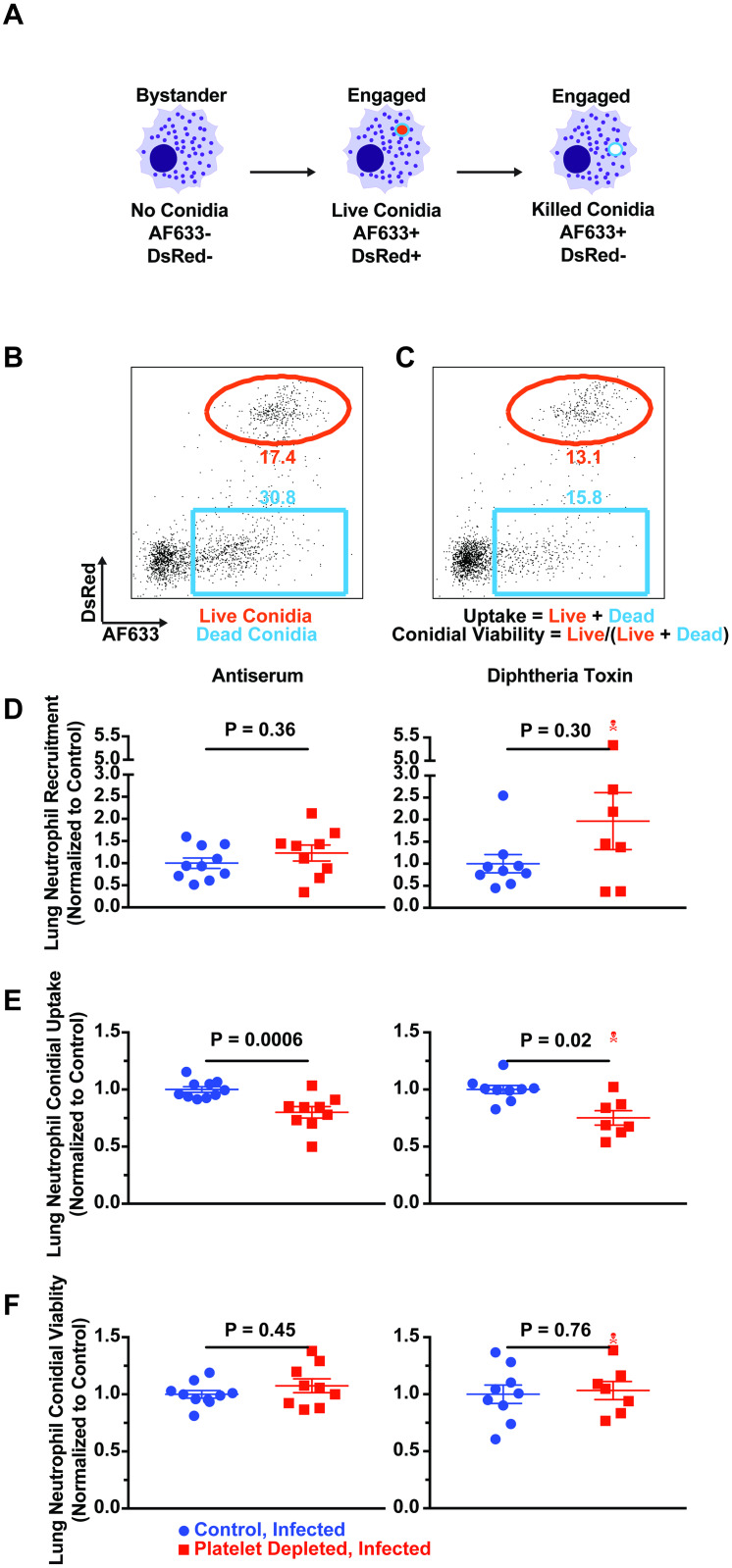
Platelets regulate neutrophil phagocytosis during *A*. *fumigatus* infection. (A) Scheme to monitor leukocyte conidial uptake and killing with FLARE conidia that express the viability fluorophore DsRed and are labeled with the tracer fluorophore Alexa Fluor 633 (AF633). Bystander lung leukocytes are DsRed^-^ and AF633^-^. Upon engulfing live FLARE conidia, leukocytes become DsRed^+^ and AF633^+^. After killing phagocytosed conidia, leukocytes are DsRed^-^ and AF633^+^. (B-C) Representative flow cytometry plots of Ly6G^+^ neutrophils analyzed for DsRed and AF633 fluorescence. The red gates indicate the frequency of neutrophils that contain live conidia, and the blue gates indicate the frequency of neutrophils that contain killed conidia in (B) control and (C) thrombocytopenic mice. (D-F) Neutrophil recruitment (D), lung neutrophil conidial uptake (E) and lung neutrophil conidiacidal activity (F) in the AS model of thrombocytopenia (left) and DT model of thrombocytopenia (right). All data are expressed as relative values, with control mice (blue) having a value of 1.00, and thrombocytopenic mice (red) as a percentage of controls at 12 hours post infection. Error bars are expressed with mean ± SEM. The skull symbol indicates a mouse died prior to harvest (excluded from analysis). Data are pooled from 2 experiments (*n* = 8–10 mice per group).

Prior studies reported that platelets regulate neutrophil recruitment to sites of inflammation [25]. Thus, we examined whether thrombocytopenia is associated with defective innate immune cell recruitment in the context of respiratory *A*. *fumigatus* infection. In both models of thrombocytopenia, platelets were dispensable for neutrophil migration to the lung when infected with Af293 conidia, with the thrombocytopenic mice having 1.23 (± 0.18) and 1.97 (± 0.65)-fold higher neutrophil counts in the AS and DT models respectively. This difference did not reach statistical significance (Figs 3D and S5A). These data suggest neutrophils are recruited to the lung in a platelet-independent manner during *A*. *fumigatus* infection.

Since platelets may enhance microbial phagocytosis and the antimicrobial activity of leukocytes [21], we next assessed the ability of lung neutrophils to phagocytose and kill conidia in the absence and presence of platelets. We inoculated thrombocytopenic or control mice with FLARE conidia, a DsRed (a conidial viability marker) transgenic Af293 strain labeled with Alexa Fluor 633 (a tracer fluorophore that does not degrade upon conidial killing) (Fig 3A) [23]. These conidia allow discrimination of bystander leukocytes, fungus-engaged leukocytes with live conidia, and fungus-engaged leukocytes with killed conidia, at single cell resolution by flow cytometry (Fig 3B and 3C). Neutrophil conidial uptake was reduced to 80% (± 5%) of controls in the AS model, and 75% of controls (± 6%) in the DT model (Fig 3E). These data indicate that lung neutrophils display a modest defect in conidial uptake in thrombocytopenic mice compared to platelet-sufficient littermate controls. In contrast, neutrophil conidial killing was similar in both thrombocytopenic and control mice (1.08 ± 0.06-fold higher in the AS model, and 1.03 ± 0.08 higher in the DT model), suggesting that platelets are dispensable for neutrophil conidiacidal activity (Fig 3F). By 40 hours post infection, the neutrophil phagocytic defect was no longer observed in thrombocytopenic mice (S6 Fig), indicating that impaired neutrophil fungal uptake was a transient defect.

### Platelets do not regulate monocyte or monocyte-derived dendritic cell (Mo-DC) function during *A*. *fumigatus* infection

Given the transient nature of the neutrophil phagocytic defect, we assessed the contribution of platelets to monocyte and Mo-DC function, since monocytes and Mo-DCs are required for host defense and enhance neutrophil function during *A*. *fumigatus* challenge [26]. In contrast to the phagocytic defect observed for neutrophils in Fig 3, no consistent differences in monocyte or Mo-DC recruitment, phagocytosis, or killing were noted in thrombocytopenic mice (S3–S5 Figs). Together, these data suggest that platelets are dispensable for the antifungal activities of monocytes and Mo-DCs.

### Syk signaling in the megakaryocytic lineage promotes neutrophil conidial uptake

**Fig 4:**
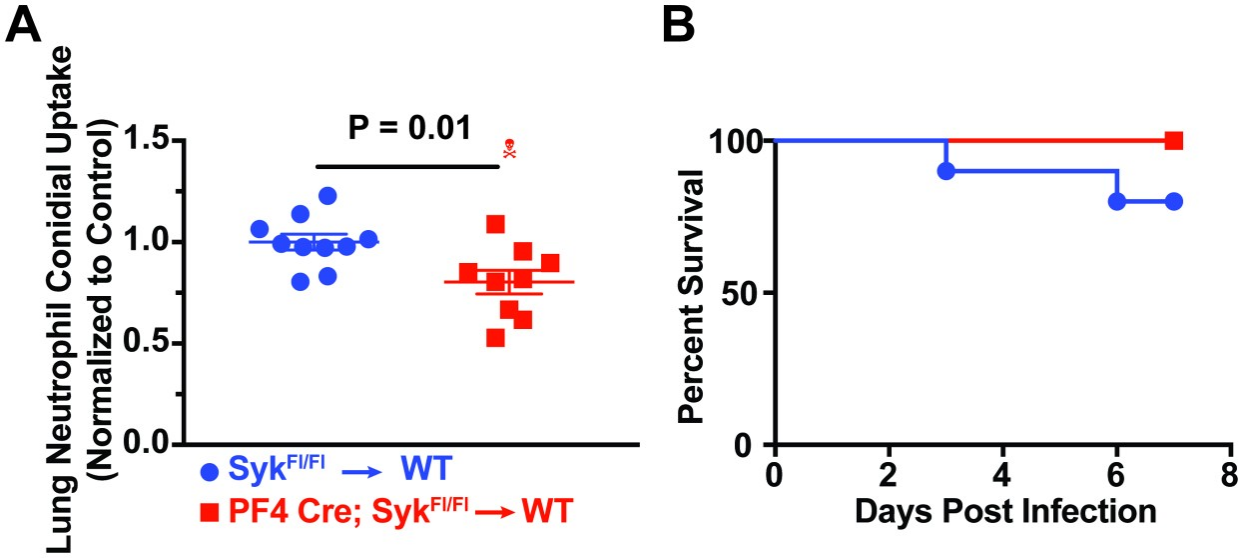
Platelet/Megakaryocyte Syk expression regulates neutrophil conidial phagocytosis but is dispensable for survival. (A) Lung neutrophil conidial uptake in bone marrow chimeric mice (Syk^ΔPf4^ → CD45.1 or Syk^fl/fl^ → CD45.1). All data are expressed as relative values, with control mice (blue) having a value of 1.00, and Syk-deficient mice (red) as a percentage of controls at 12 hours post infection. Error bars are expressed with mean ± SEM. The skull symbol indicates a mouse died prior to harvest (excluded from analysis). Data are pooled from 2 separate experiments (*n* = 10 mice per group). (B) Kaplan-Meier survival curve of bone marrow chimeric mice (Syk^ΔPf4^ → CD45.1 or Syk^fl/fl^ → CD45.1) infected with ~3-6x10^7^ *A*. *fumigatus* CEA10 conidia. Data are pooled from 2 experiments (*n* = 9–10 mice per group).

Conidial germination results in surface exposure of β-glucan polysaccharides that trigger innate immune signaling through Dectin-1, Syk, and CARD9 [27]. Previously, we reported that genetic defects in the Dectin-1/Syk/CARD9 signaling pathway leads to impaired conidial phagocytosis by neutrophils [28]. Because Syk integrates signaling from C-type lectin and integrin receptors expressed both on leukocytes [28] and platelets [29], we deleted Syk in Pf4-expressing cells, resulting in a loss of platelet Syk expression (S7 Fig). We then assessed the role of platelet/megakaryocyte Syk signaling in neutrophil conidial phagocytosis by infecting Syk^ΔPf4^ chimeras, or Cre-negative Syk^fl/fl^ control chimeras with FLARE conidia. Conditional Syk deletion in Pf4^+^ cells reduced neutrophil conidial uptake to 80% (± 6%) of Syk-sufficient controls (Fig 4A). This result suggests that Syk expression in platelets regulates neutrophil conidial phagocytosis in a manner similar to complete platelet depletion.

To assess the contribution of megakaryocytic lineage Syk signaling to fungal susceptibility, we challenged Syk^ΔPf4^ chimeras, or Cre-negative Syk^fl/fl^ control chimeras with CEA10 conidia. There is no significant difference in survival among both groups (Fig 4B). These data indicated that Syk signaling in megakaryocytes and platelets and its role in mediating neutrophil conidial phagocytosis were not responsible for the mortality phenotype seen in both the AS and DT models of thrombocytopenia.

### Thrombocytopenia leads to pulmonary hemorrhage and tissue damage after *A*. *fumigatus* challenge

**Fig 5:**
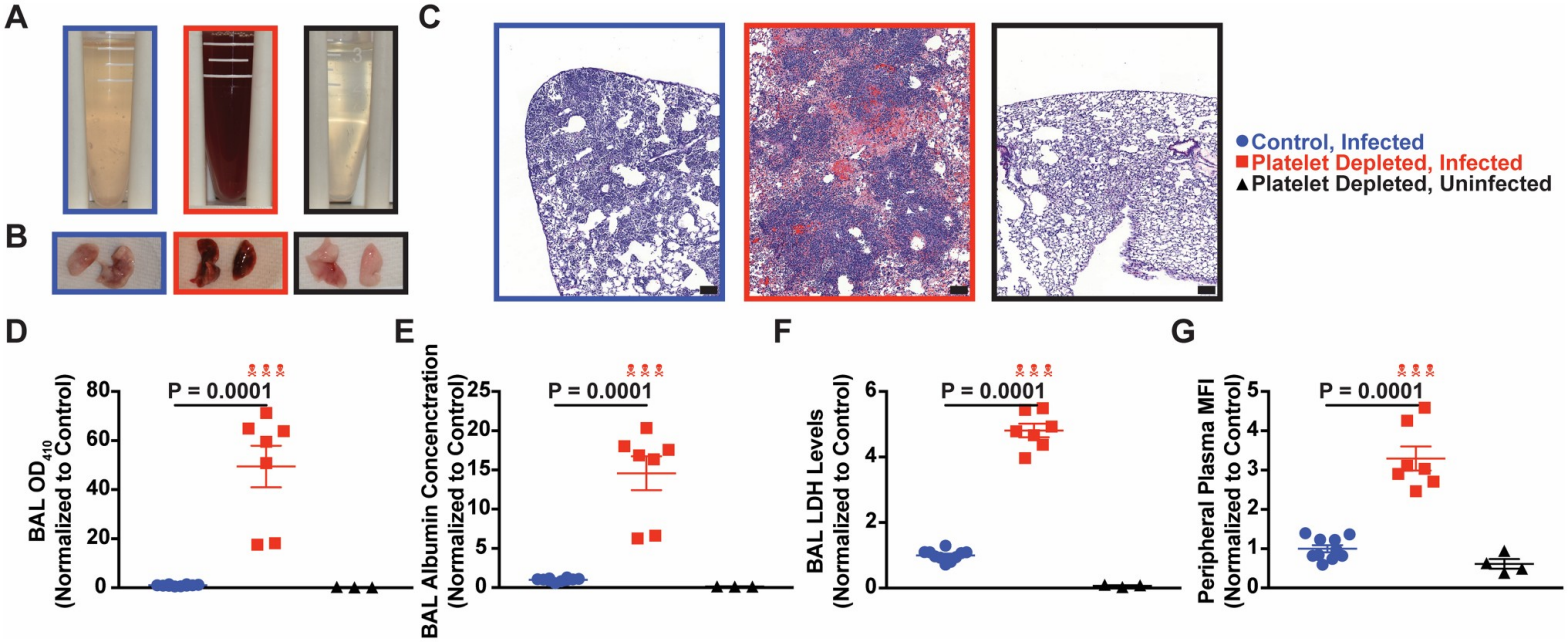
Thrombocytopenia impairs lung tissue integrity after *Aspergillus* challenge. (A) Representative images of bronchoalveolar lavage fluid (BALF), (B) whole perfused lungs, (C) H&E stained lung sections (scale bar: 100 μm, images are representative of 4–5 mice per group), (D) BALF OD_410_ absorption measurements, (E) BALF albumin levels, and (F) BALF LDH levels 2 days after infection of platelet sufficient mice infected with ~3–6 x 10^7^ *A*. *fumigatus* CEA10 conidia (blue), thrombocytopenic (AS model) mice infected (red) or uninfected thrombocytopenic mice (black). (G) Vascular permeability assessed by monitoring peripheral plasma fluorescence after intranasal installation of FITC-Dextran prior to euthanasia. All data are expressed as relative values, with control mice (blue) having a value of 1.00, and thrombocytopenic mice (red) and uninfected thrombocytopenic mice (black) as a percentage of infected controls at approximately 48 hours post infection. Error bars are expressed with mean ± SEM. The skull symbol indicates a mouse died prior to harvest (excluded from analysis). Data from (D-G) are pooled from 2 experiments for infected mice (*n* = 10 mice per group), and 1 experiment for uninfected mice (*n* = 3–4 mice per group).

Since functional defects in neutrophil phagocytosis did not explain the mortality phenotype in thrombocytopenic mice, we next explored the contribution of platelets to lung tissue integrity. Since thrombocytopenia can promote pulmonary hemorrhage after instillation of inflammatory stimulus (e.g., LPS [30]), we harvested bronchoalveolar lavage fluid (BALF) and lung tissue after CEA10 infection to qualitatively assess pulmonary hemorrhage and tissue damage. We found that in both the AS (Fig 5A and 5B) and DT models (S11A and S11B Fig) of thrombocytopenia, a loss of platelets led to severe hemorrhage in the airways and lung parenchyma. Hemorrhage was readily evident in thrombocytopenic AS model mice compared with the control mice in H&E stained lung sections (Figs 5C and S8–S10). We found a significant increase in hemorrhage in thrombocytopenic infected mice compared to platelet-sufficient controls as assessed by BALF OD_410_ absorbance measurements. Thrombocytopenic mice exhibited a 49.44 (± 8.48)-fold higher OD_410_ reading at 48 hours post infection in the AS model, and a 34.46 (± 12.12)-fold higher OD_410_ reading at 24 hours post infection in the DT model (Figs 5D and S11C). These data demonstrate that platelets maintain hemostasis in the lung during respiratory fungal infection.

In agreement with these findings, a BCG assay found an increase in albumin levels in the BALF of infected thrombocytopenic animals compared to infected platelet-sufficient control mice, with a 14.58 (± 2.15)-fold increase in the AS model, and a 12.74 (± 3.81)-fold increase in the DT model (Figs 5E and S11D). Similarly, we found an increase in the BALF lactate dehydrogenase (LDH) level in infected thrombocytopenic mice compared with infected platelet-sufficient controls, with a 4.81 (± 0.21)-fold increase in the AS model, and 6.45 (± 1.50)-fold increase in the DT model. (Figs 5F and S11E). Thus, the presence of platelets reduced lung tissue damage during respiratory *Aspergillus* infection.

Given the presence of blood in the airways, we assessed the permeability of the lung airway barrier by assessing diffusion of FITC-dextran particles from the airways to the vasculature. We found a significant increase in FITC fluorescence in peripheral plasma (Figs 5G and S11F), suggesting a breakdown in the barrier between the airways and the blood in the absence of platelets (3.30 (± 0.31)-fold higher in the AS model, and 2.36 (± 0.40)-fold higher in the DT model). As expected from the survival data (Fig 1), uninfected thrombocytopenic mice had no evidence of hemorrhage or tissue damage (Fig 5A–5G).

To assess the contribution of fungal germination to lung tissue damage, we quantified the conidial germination rate in the AS model on GMS-stained lung sections. We did not observe a statistically significant difference in fungal germination in the lungs of thrombocytopenic mice compared to the lungs of control mice (S12A–S12D Fig). We did not observe overt evidence of fungal angioinvasion in either group, consistent with the model that inflammatory lung hemorrhage was the primary cause of mortality in thrombocytopenic mice.

### Fungal germination is required for mortality in thrombocytopenic mice

**Fig 6:**
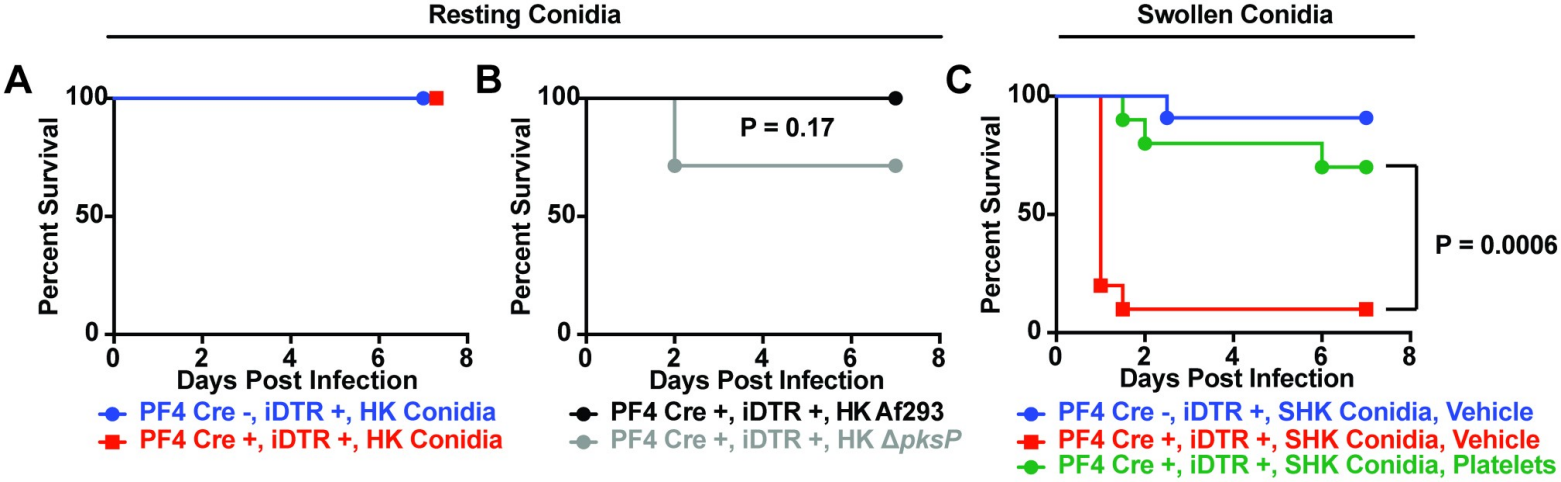
Fungus-induced inflammation drives mortality in the diphtheria toxin model of thrombocytopenia. (A) Kaplan-Meier survival curve of Cre negative (blue) or iDTR^Pf4^ (red) mice infected with ~6x10^7^ resting, heat-killed CEA10 conidia. Data are pooled from 2 experiments (*n* = 8–12 mice per group). (B) Kaplan-Meier survival curve of iDTR^Pf4^ mice infected with ~6 x 10^7^ resting, heat-killed, WT Af293 (black) or a Δ*pksP* mutant strain of Af293 (grey). Data are pooled from 2 experiments (*n* = 6–7 mice per group). (C) Kaplan-Meier survival curve of Cre negative (blue) or iDTR^Pf4^ (red, green) mice infected with ~6x10^7^ swollen, heat-killed CEA10 conidia. 1 hour after infection, mice were injected with 1 x 10^8^ platelets (green) or Tyrode’s buffer supplemented with BSA and glucose (blue, red). Data are pooled from 3 experiments (*n* = 10–11 mice per group).

To determine whether fungal germination and viability were necessary to induce mortality in thrombocytopenic mice, we compared murine survival in thrombocytopenic mice challenged with heat-killed resting conidia and heat-killed swollen conidia, the first step in germination and hyphal growth. Inoculation with resting heat-killed conidia failed to induce any mortality in either thrombocytopenic (0/8) or control (0/12) mice in the DT model (Fig 6A). This suggests that fungal germination or metabolic activity is necessary to induce inflammation-dependent mortality in thrombocytopenic mice.

In resting conidia, the rodlet and melanin layers mask the internal immunogenic polysaccharides, primarily β-glucans [31,32]. A mutant deficient in melanin synthesis (Δ*pksP*) has exposed β-glucans in the resting state [33]. Infection of thrombocytopenic mice with heat-killed resting Δ*pksP* conidia killed a portion of thrombocytopenic mice in the DT model (2/7 compared to 0/6, P = 0.17), but did not completely recapitulate the survival defect seen in live infections with resting wild-type conidia (Fig 6B). This result suggested that the surface exposure of cell wall carbohydrates in amelanotic resting conidia is not fully sufficient to induce lung hemorrhage and death observed in thrombocytopenic hosts infected with live wild-type conidia that have the capacity to germinate.

During conidial swelling, fungal cells lose their melanin and hydrophobin layers and increase in diameter from ~2 to ~4 μm, and thereby increase the amount (through de novo synthesis) and exposure of immunoactive β-glucan carbohydrates [34]. Thrombocytopenic mice (DT model) were highly susceptible (90%) to challenge with heat-killed swollen conidia compared with platelet-sufficient controls (9%; Fig 6C). This finding indicated that fungal germination-induced inflammation, but not fungal growth per se, resulted in lethal lung hemorrhage in thrombocytopenic mice.

This model predicts that platelet transfusion should reverse the susceptibility of platelet depletion followed by challenge with swollen, heat-killed conidia. To test this conjecture, we transfused 1 x 10^8^ platelets 1 hour after inoculation of heat-killed swollen conidia in thrombocytopenic mice (DT model). Following platelet transfusion, murine mortality was severely mitigated and only 3 of 10 transfused mice succumbed to this challenge (Fig 6C). This finding suggested that a low level of platelets is sufficient to overcome pathologic lung inflammation and confirmed the tissue-protective role of platelets in this model.

## Discussion

In this study, we established a critical, non-redundant role for platelets in host defense and lung tissue integrity during respiratory *A*. *fumigatus* infection. We observed that platelet loss caused susceptibility to pulmonary *A*. *fumigatus* challenge independent of the fungal strain used and independent of the platelet depletion method. Murine mortality during thrombocytopenia was coincident with a reduction in neutrophil conidial phagocytosis; this phenotype was mediated by megakaryocyte lineage Syk signaling. However, the platelet-dependent neutrophil conidial phagocytosis defect was not severe and did not contribute to mortality, and conditional deletion of Syk in the megakaryocyte lineage did not decrease survival. Instead, infection of thrombocytopenic animals led to severe lung hemorrhage in the absence of a marked increase in hyphal growth. Thrombocytopenic mice were susceptible to inoculation with highly inflammatory heat-killed swollen conidia, but not immunologically inert heat-killed resting conidia. Taken together, our results indicate that platelets primarily function during aspergillosis to maintain lung hemostasis in response to inflammatory stimulus triggered by conidial germination.

The ability of platelets to protect hosts against bacterial pathogens is well documented. Platelets are protective against intravenous *Staphylococcus aureus* infection, and thrombocytopenic mice have a higher kidney bacterial burden in a DT model of thrombocytopenia [35]. In an antibody-mediated model of thrombocytopenia, mice were vulnerable to pulmonary *Pseudomonas aeruginosa* infection, which is coincident with higher bacterial lung CFUs [36]. Our results extend these prior studies to a medically relevant fungal pathogen.

Platelets have been reported to aid in leukocyte recruitment to sites of inflammation [37]. In our study, *A*. *fumigatus* infection induces neutrophil recruitment independent of the presence or absence of platelets, suggesting redundancies in the cellular sources of neutrophil recruitment cues that likely include platelet-independent production of CXCL1, CXCL2, CXCL5 [28], IL-1α [38] and LTB_4_ [39]. This finding contrasts with reports that platelets mediate innate immune cell recruitment conducted in both live bacterial infections and sterile inflammation [36,40–42], but is in line with another report that documents platelet-independent innate immune cell recruitment [12]. Notably, none of these studies utilized live fungi or fungal ligands.

Beyond neutrophil recruitment, platelets have been reported to mediate leukocyte anti-fungal effector functions in vitro [13]. Specifically, a study reported that addition of platelet-rich plasma improved macrophage conidial phagocytosis, and enhanced macrophage fungistatic activity, as measured by a reduction in fungal metabolic activity [21]. Our in vivo data indicated that platelets enhanced neutrophil conidial phagocytosis in a Syk-dependent manner, since complete loss of platelets or loss of platelet-specific Syk expression reduced neutrophil conidial uptake. However, our data did not support a role for platelets in conidial killing by lung-infiltrating neutrophils. These results likely reflect important differences between in vitro and in vivo experimental approaches, including numeric differences in effector cell to fungal cell ratios found in the test tube and lung as well as functional differences between lung-infiltrating neutrophils and cultured macrophages in the test tube. Though our in vivo data do not offer a mechanism for the differential fungal burden given the transient nature of the neutrophil defect and normal monocyte and Mo-DC activity, published in vitro studies [14–19] are consistent with a role for platelets in direct anti-fungal activity instead.

During bacterial inflammatory challenges, platelets are reported to maintain hemostasis and tissue integrity. Instillation of LPS into the airways of thrombocytopenic mice results in severe hemorrhage [30,43], as does pulmonary infection with *Klebsiella pneumoniae* [12]. In agreement with these findings, we observed a breakdown in lung tissue integrity and hemorrhage into the airways and lung parenchyma. As we saw limited hyphal formation after infection with live conidia, and high mortality after inoculation with swollen, heat-killed, conidia, our data strongly suggest that there is no strict requirement for hyphal angioinvasion to induce tissue damage and murine mortality in thrombocytopenic *A*. *fumigatus*-infected mice. Fungal-induced inflammation per se caused sufficient vascular permeability and lung hemorrhage to kill mice. This model is in agreement with the finding that killed hyphae can induce endothelial injury in vitro [44], implying that *A*. *fumigatus* germination is harmful under specific host conditions, such as thrombocytopenia.

The exact molecular mechanism of platelet mediated hemostasis during infection remains an open question. In contrast to multiple reports implicating the Syk-coupled platelet receptors GPVI [9] and CLEC-2 [10] in maintaining vascular integrity in response to sterile inflammation [45,46], we did not observe a mortality defect in platelet/megakaryocyte Syk-deficient chimeric mice in response to infection. However, another study reported that depleting GPVI or CLEC-2 did not cause severe hemorrhage in response to *K*. *pneumoniae* infection [47]. The authors suggested that differential regulation of hemostasis in sterile inflammation and during bacterial infection may account for these discrepancies. This explanation is in agreement with our data, since swollen, heat-killed conidia induced similar levels of inflammation in the lung as live resting conidia that undergo swelling prior to killing by lung-infiltrating neutrophils and macrophages [34].

Hemoptysis is a symptom of invasive aspergillosis which has the potential to kill patients despite recent advances in prophylaxis and antifungal therapies [48]. However, when IA occurs in patients with chemotherapy-induced neutropenia, hemoptysis is more common and is often associated with a rebound in neutrophil counts [49]. In this group, neutropenia and thrombocytopenia are usually concurrent [6], and baseline platelet counts stratify neutropenia-related IA outcomes into high- and low-risk categories [4]. Because neutrophil recruitment associated with bacterial inflammation induced pulmonary hemorrhage during thrombocytopenia [43], the potential pathological impact of neutrophil recovery and infiltration in thrombocytopenic hosts remains an important question for future research. Beyond the potential pathogenic role of neutrophils, IL-1R signaling and CXCR2 signaling are critical for anti-*Aspergillus* host defense [28]. These receptors are implicated in vascular permeability [50,51]. Future studies will clarify whether platelets or platelet-derived products regulate these signaling pathways to prevent uncontrolled activation and immune pathology during the early lung response to *A*. *fumigatus*.

Our work defines a major role for platelets in the maintenance of lung tissue integrity during pulmonary *A*. *fumigatus* challenge that is largely independent of hyphal tissue invasion. Although platelets played a minor role in innate immune regulation in the lung, this effect was dispensable for host survival. Since platelet transfusion improved murine outcomes, further work may reveal a beneficial therapeutic role for platelets in human patients at risk for, or diagnosed with IA.

## Supporting information

S1 Fig Absolute platelet counts in thrombocytopenic and control mice. (A) Peripheral blood platelet counts in C57BL/6 mice injected with control serum (blue) or platelet targeting antiserum (red). (B) Peripheral blood platelet counts in uninjected C57BL/6 mice infected with ~3x10^7^ CEA10 conidia. (C) Peripheral blood platelet counts in iDTR^Pf4^ or Cre-negative littermates injected with DT on days 0, 2, and 4.
(TIF)

S2 Fig Platelets protect mice in a fungal strain-independent manner. Kaplan-Meier survival curve of C57BL/6J mice treated with normal rabbit serum (blue) or platelet targeting serum (red), and infected with ~6x10^7^ *A*. *fumigatus* Af293 conidia. Data are from one experiment (*n* = 5 mice per group).
(TIF)

S3 Fig Platelets do not regulate monocyte function after *A*. *fumigatus* infection. Lung monocyte recruitment (A), conidial uptake (B) and conidiacidal activity (C) in the AS model of thrombocytopenia (left) and DT model of thrombocytopenia (right). All data are expressed as relative values, with control mice (blue) having a value of 1.00, and thrombocytopenic mice (red) as a percentage of controls at 12 hours post infection. Error bars are expressed with mean ± SEM. The skull symbol indicates a mouse died prior to harvest (excluded from analysis). Data are pooled from 2 experiments (*n* = 8–10 mice per group). For comparisons between two groups, a Mann-Whitney U test was used.
(TIF)

S4 Fig Platelets do not regulate Mo-DC function after A. fumigatus infection. Lung Mo-DC recruitment (A), conidial uptake (B) and conidiacidal activity (C) in the AS model of thrombocytopenia (left) and DT model of thrombocytopenia (right). All data are expressed as relative values, with control mice (blue) having a value of 1.00, and thrombocytopenic mice (red) as a percentage of controls at 12 hours post infection. Error bars are expressed with mean ± SEM. The skull symbol indicates a mouse died prior to harvest (excluded from analysis). Data are pooled from 2 experiments (*n* = 8–10 mice per group). For comparisons between two groups, a Mann-Whitney U test was used.
(TIF)

S5 Fig Absolute lung cell counts for neutrophils, monocytes and Mo-DCs during *A*. *fumigatus* infection. Lung neutrophil (A), monocyte (B), and monocyte-derived dendritic cell (C) recruitment in platelet sufficient (blue) or thrombocytopenic (red) mice 12 hours after infection. Error bars are expressed with mean ± SEM. The skull symbol indicates a mouse died prior to harvest (excluded from analysis). Data are pooled from 2 experiments (*n* = 8–10 mice per group). For comparisons between two groups, a Mann-Whitney U test was used.
(TIF)

S6 Fig Platelet regulate lung neutrophil phagocytosis transiently. Neutrophil recruitment (A), lung neutrophil conidial uptake (B) and lung neutrophil conidiacidal activity (C) in the AS model of thrombocytopenia (left) and DT model of thrombocytopenia (right). All data are expressed as relative values, with control mice (blue) having a value of 1.00, and thrombocytopenic mice (red) as a percentage of controls at 40 hours post infection. Error bars are expressed with mean ± SEM. The skull symbol indicates a mouse died prior to harvest (excluded from analysis). Data are pooled from 2 experiments (*n* = 9–13 mice per group). For comparisons between two groups, a Mann-Whitney U test was used.
(TIF)

S7 Fig PF4-iCre efficiently deletes Syk in platelets. Western blot of increasing amounts of platelet lysate obtained from Syk^fl/fl^ control mice (left) or platelet lysate from Syk^ΔPf4^ mice (right). The upper band (green) shows Syk expression, and the lower band (red) shows β-actin as a loading control. Platelets were pooled from 2 mice.
(TIF)

S8 Fig Lung histopathology from platelet-sufficient infected mice, related to Fig 5. Full H&E stained sections at 1x (A) and 10x (B) magnification from Fig 5C. The black box in the 1x section indicates the location of the 10x image in this figure and in Fig 5C.
(PDF)

S9 Fig Lung histopathology from thrombocytopenic infected mice, related to Fig 5. Full H&E stained sections at 1x (A) and 10x (B) magnification from Fig 5C. The black box in the 1x section indicates the location of the 10x image in this figure and in Fig 5C.
(PDF)

S10 Fig Lung histopathology from thrombocytopenic uninfected mice, related to Fig 5. Full H&E stained sections at 1x (A) and 10x (B) magnification from Fig 5C. The black box in the 1x section indicates the location of the 10x image in this figure and in Fig 5C.
(PDF)

S11 Fig A second model of thrombocytopenia impairs lung tissue function integrity *Aspergillus* challenge early in infection. (A) Representative images of bronchoalveolar lavage fluid (BALF) and (B) perfused lungs 1 day after infection of Cre negative (blue) or iDTR^Pf4^ (red) mice injected with DT and infected with ~3–6 x 10^7^ *A*. *fumigatus* CEA10 conidia. (C) Lung airway bleeding (from A) quantified by measuring BALF OD_410_ absorption, (D) airway vascular leakage determined by BALF albumin levels, (E) airway LDH levels, and (F) vascular permeability assessed by monitoring peripheral plasma fluorescence after intranasal installation of FITC-Dextran prior to euthanasia in thrombocytopenic and control mice. All data are expressed as relative values, with control mice (blue) having a value of 1.00, and thrombocytopenic mice (red) as a percentage of controls at approximately 24 hours post infection. Error bars are expressed with mean ± SEM. The skull symbol indicates a mouse died prior to harvest (excluded from analysis). Data from (C-F) are pooled from 2 experiments (*n* = 7–9 total mice per group).
(TIF)

S12 Fig The fungal burden in thrombocytopenic mice is not coincident with an increase in fungal germination. (A-B) Representative GMS staining of control infected (blue, A), or thrombocytopenic infected (red, B), C56BL/6J mouse lung 2 days after infection with ~3–6 x 10^7^ *A*. *fumigatus* CEA10 conidia in the AS model. The scale bars represent 50 μm. (C) Quantification of the germination rate in control infected (blue) and thrombocytopenic infected (red) C57BL/6J mice. Error bars are expressed with mean ± SEM. The skull symbol indicates a mouse died prior to harvest and was excluded from analysis. Data are from one experiment (*n* = 5 mice per group). (D) Germination rate in C was determined by counting all fungal particles and by dividing the number of germlings and hyphae by the total number.
(TIF)
