# Supplementary figures and images for: Platelets are critical for survival and tissue integrity during murine pulmonary Aspergillus fumigatus infection
Source: PLoS Pathog. 2020 May 14;16(5):e1008544. doi: 10.1371/journal.ppat.1008544 (PMC7252636; doi:10.1371/journal.ppat.1008544)

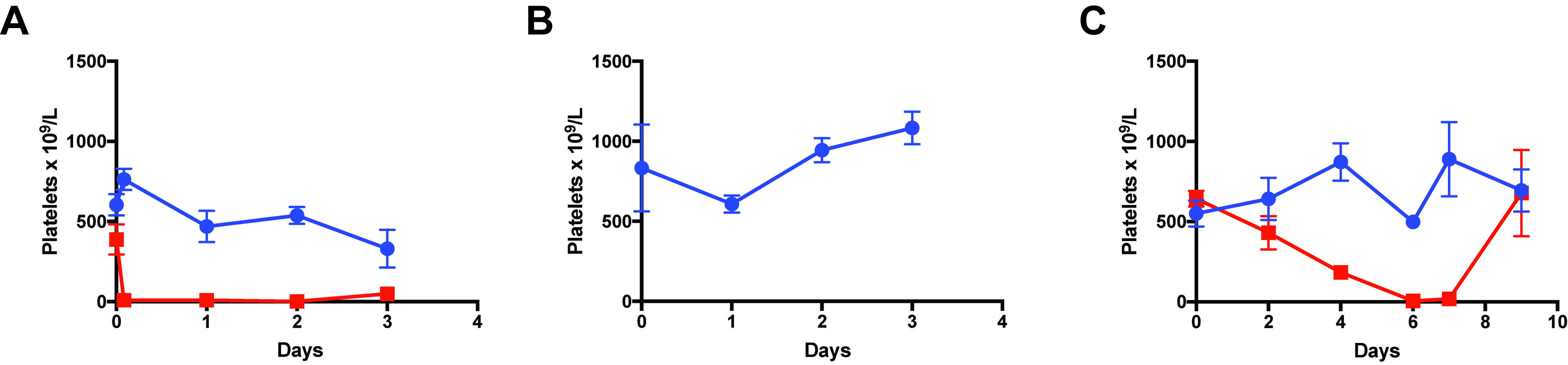

Supplement: S1 Fig — (A) Peripheral blood platelet counts in C57BL/6 mice injected with control serum (blue) or platelet targeting antiserum (red). (B) Peripheral blood platelet counts in uninjected C57BL/6 mice infected with ~3x10^7^ CEA10 conidia. (C) Peripheral blood platelet counts in iDTRPf4 or Cre-negative littermates injected with DT on days 0, 2, and 4. (TIF) [file ppat.1008544.s001.tif]

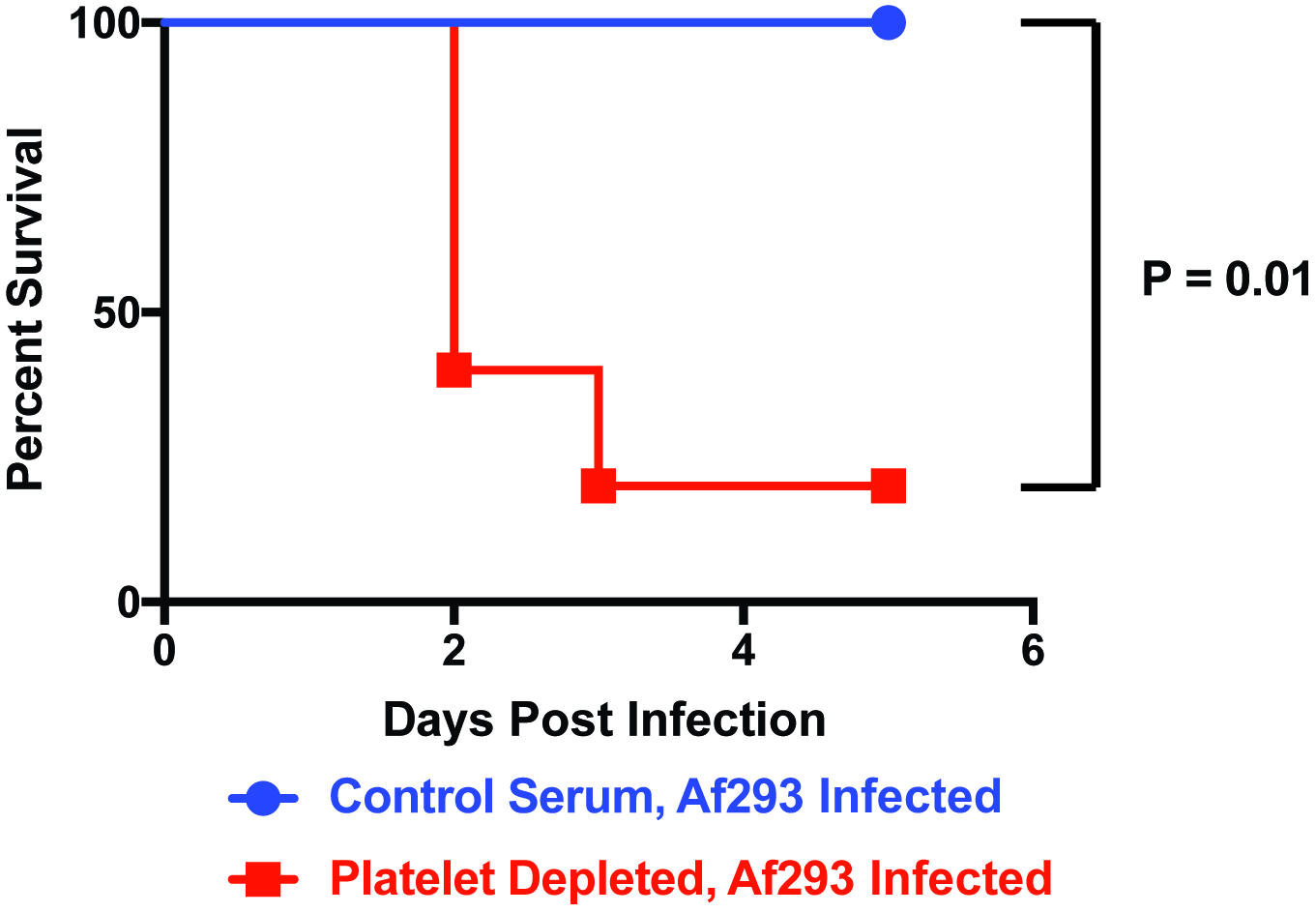

Supplement: S2 Fig — Kaplan-Meier survival curve of C57BL/6J mice treated with normal rabbit serum (blue) or platelet targeting serum (red), and infected with ~6x10^7^ A. fumigatus Af293 conidia. Data are from one experiment (n = 5 mice per group). (TIF) [file ppat.1008544.s002.tif]

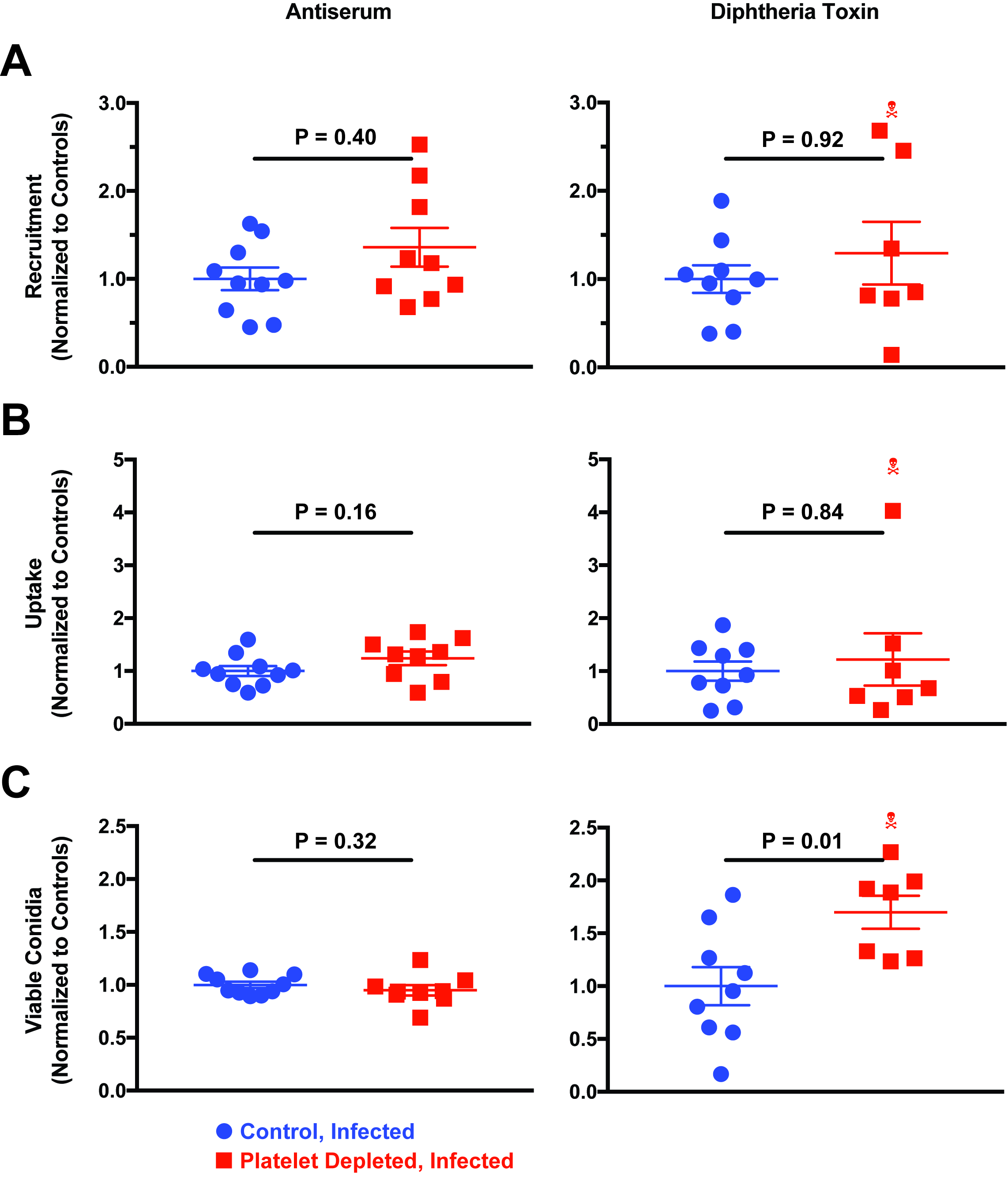

Supplement: S3 Fig — Lung monocyte recruitment (A), conidial uptake (B) and conidiacidal activity (C) in the AS model of thrombocytopenia (left) and DT model of thrombocytopenia (right). All data are expressed as relative values, with control mice (blue) having a value of 1.00, and thrombocytopenic mice (red) as a percentage of controls at 12 hours post infection. Error bars are expressed with mean ± SEM. The skull symbol indicates a mouse died prior to harvest (excluded from analysis). Data are pooled from 2 experiments (n = 8–10 mice per group). For comparisons between two groups, a Mann-Whitney U test was used. (TIF) [file ppat.1008544.s003.tif]

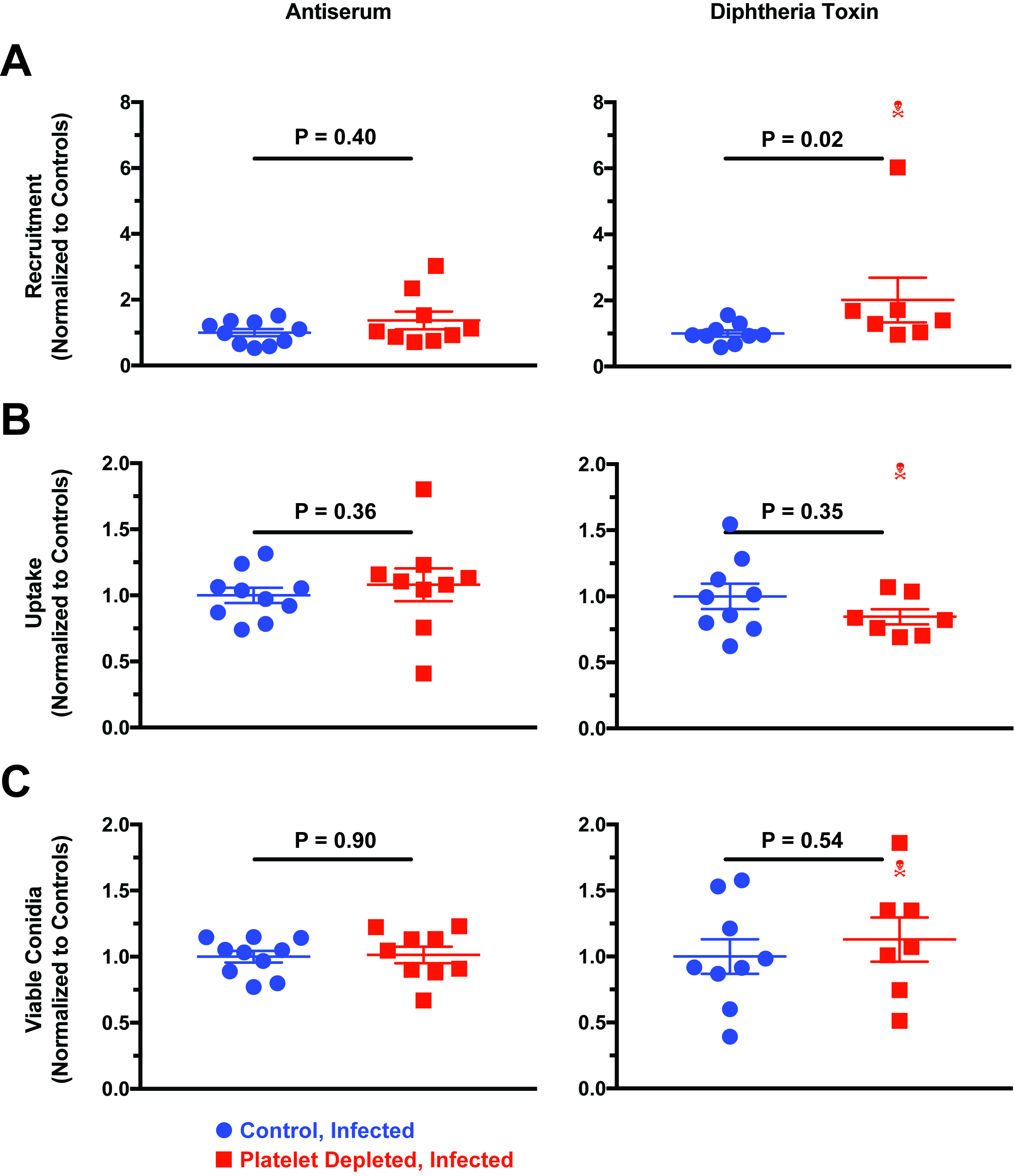

Supplement: S4 Fig — Lung Mo-DC recruitment (A), conidial uptake (B) and conidiacidal activity (C) in the AS model of thrombocytopenia (left) and DT model of thrombocytopenia (right). All data are expressed as relative values, with control mice (blue) having a value of 1.00, and thrombocytopenic mice (red) as a percentage of controls at 12 hours post infection. Error bars are expressed with mean ± SEM. The skull symbol indicates a mouse died prior to harvest (excluded from analysis). Data are pooled from 2 experiments (n = 8–10 mice per group). For comparisons between two groups, a Mann-Whitney U test was used. (TIF) [file ppat.1008544.s004.tif]

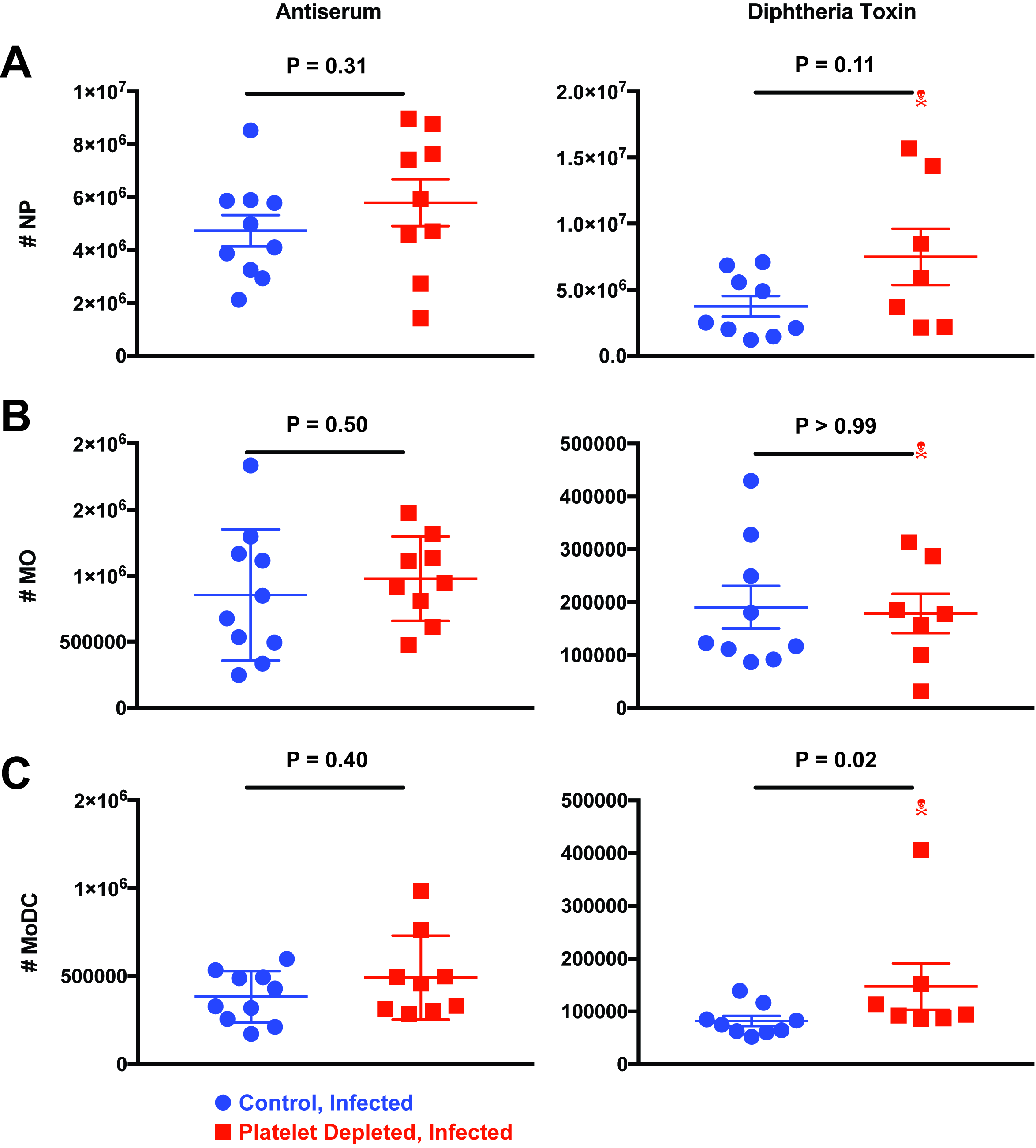

Supplement: S5 Fig — Lung neutrophil (A), monocyte (B), and monocyte-derived dendritic cell (C) recruitment in platelet sufficient (blue) or thrombocytopenic (red) mice 12 hours after infection. Error bars are expressed with mean ± SEM. The skull symbol indicates a mouse died prior to harvest (excluded from analysis). Data are pooled from 2 experiments (n = 8–10 mice per group). For comparisons between two groups, a Mann-Whitney U test was used. (TIF) [file ppat.1008544.s005.tif]

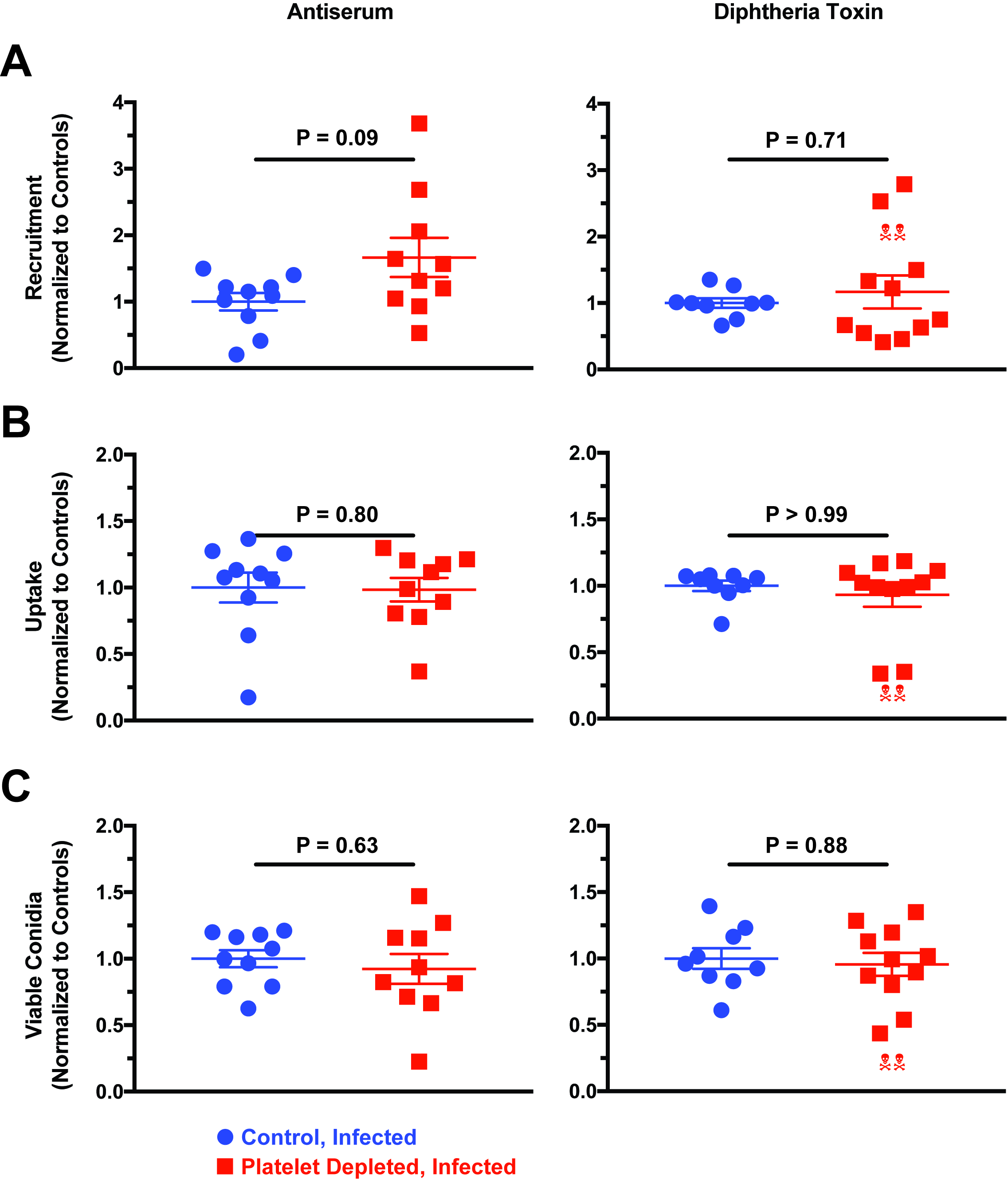

Supplement: S6 Fig — Neutrophil recruitment (A), lung neutrophil conidial uptake (B) and lung neutrophil conidiacidal activity (C) in the AS model of thrombocytopenia (left) and DT model of thrombocytopenia (right). All data are expressed as relative values, with control mice (blue) having a value of 1.00, and thrombocytopenic mice (red) as a percentage of controls at 40 hours post infection. Error bars are expressed with mean ± SEM. The skull symbol indicates a mouse died prior to harvest (excluded from analysis). Data are pooled from 2 experiments (n = 9–13 mice per group). For comparisons between two groups, a Mann-Whitney U test was used. (TIF) [file ppat.1008544.s006.tif]

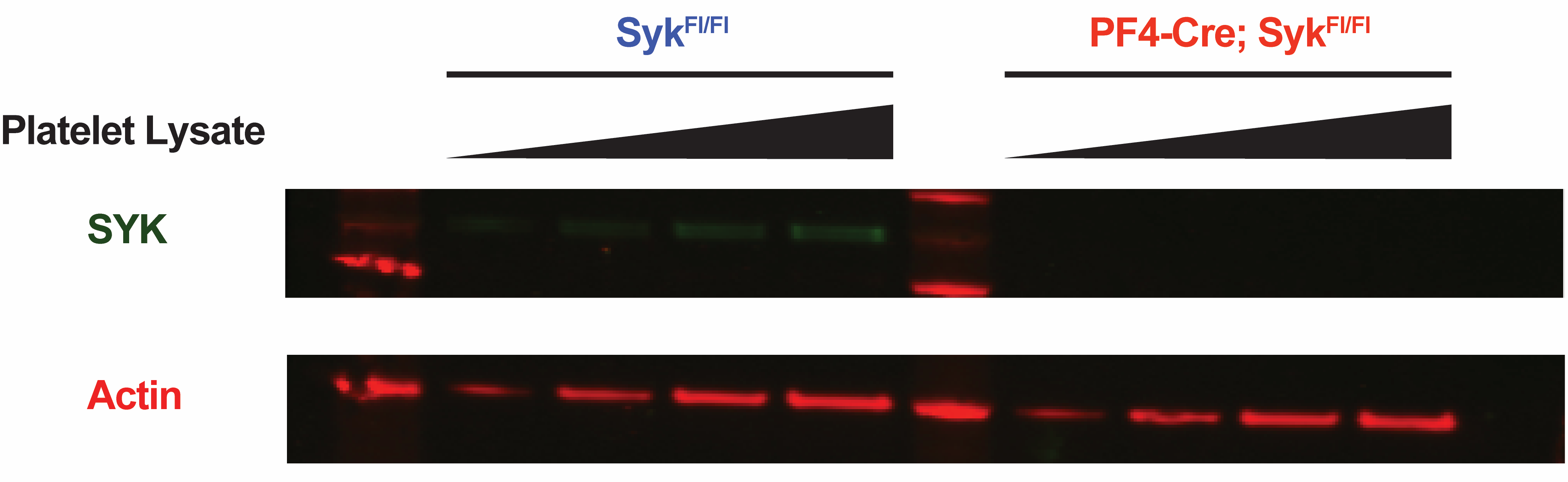

Supplement: S7 Fig — Western blot of increasing amounts of platelet lysate obtained from Sykfl/fl control mice (left) or platelet lysate from SykΔPf4 mice (right). The upper band (green) shows Syk expression, and the lower band (red) shows β-actin as a loading control. Platelets were pooled from 2 mice. (TIF) [file ppat.1008544.s007.tif]

2000  $\mu\text{m}$

A

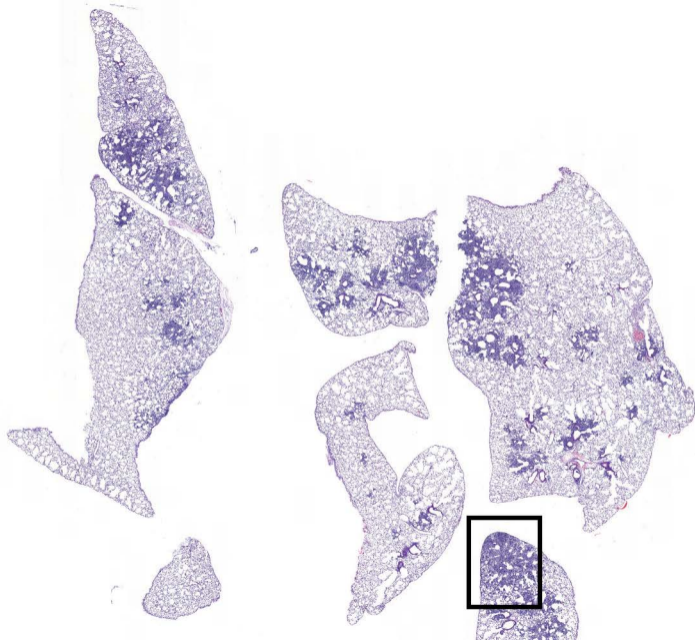

B

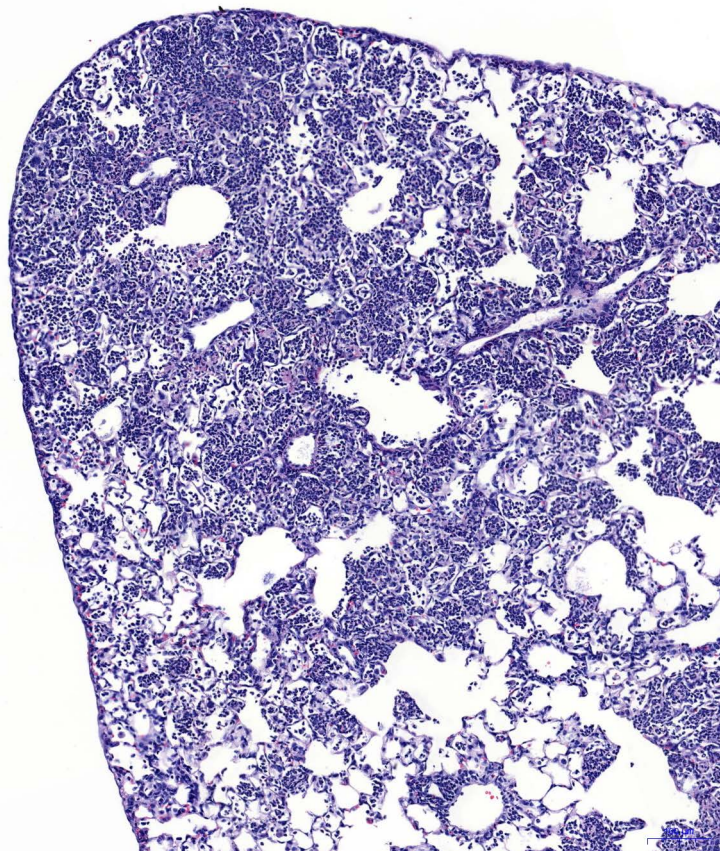

Supplement: S8 Fig — Full H&E stained sections at 1x (A) and 10x (B) magnification from Fig 5C. The black box in the 1x section indicates the location of the 10x image in this figure and in Fig 5C. (PDF) [file ppat.1008544.s008.pdf]

2000  $\mu\text{m}$

A

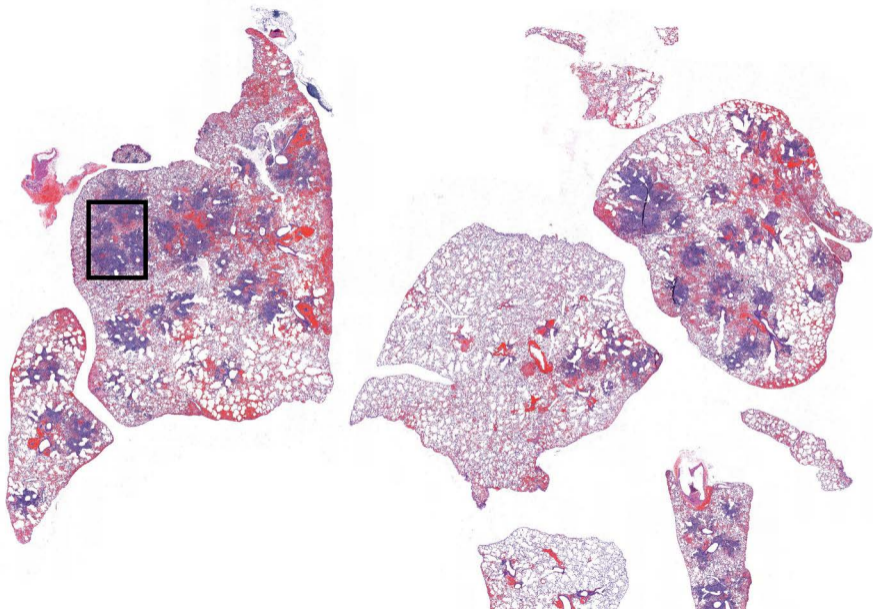

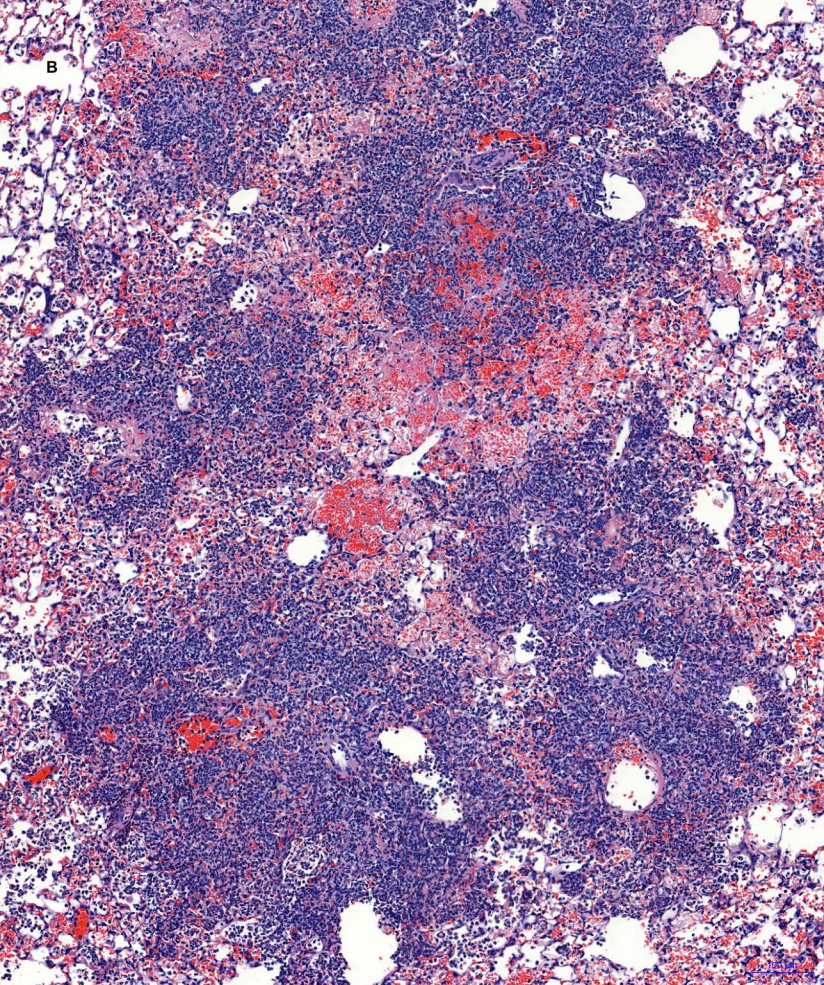

Supplement: S9 Fig — Full H&E stained sections at 1x (A) and 10x (B) magnification from Fig 5C. The black box in the 1x section indicates the location of the 10x image in this figure and in Fig 5C. (PDF) [file ppat.1008544.s009.pdf]

2000  $\mu\text{m}$

A

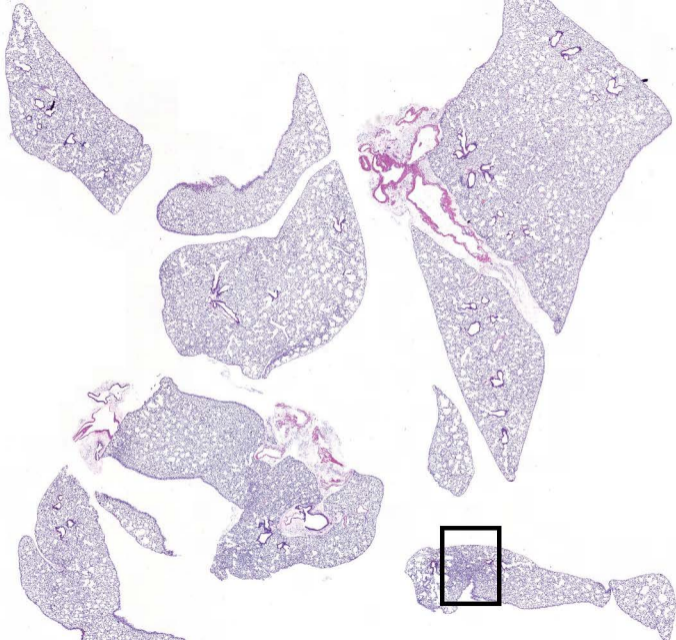

B

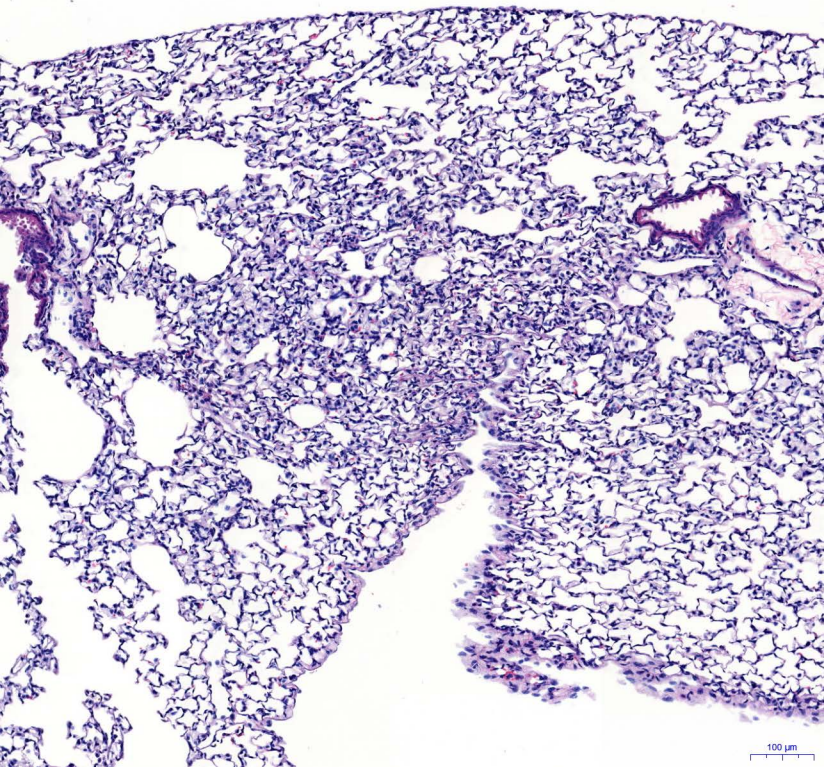

Supplement: S10 Fig — Full H&E stained sections at 1x (A) and 10x (B) magnification from Fig 5C. The black box in the 1x section indicates the location of the 10x image in this figure and in Fig 5C. (PDF) [file ppat.1008544.s010.pdf]

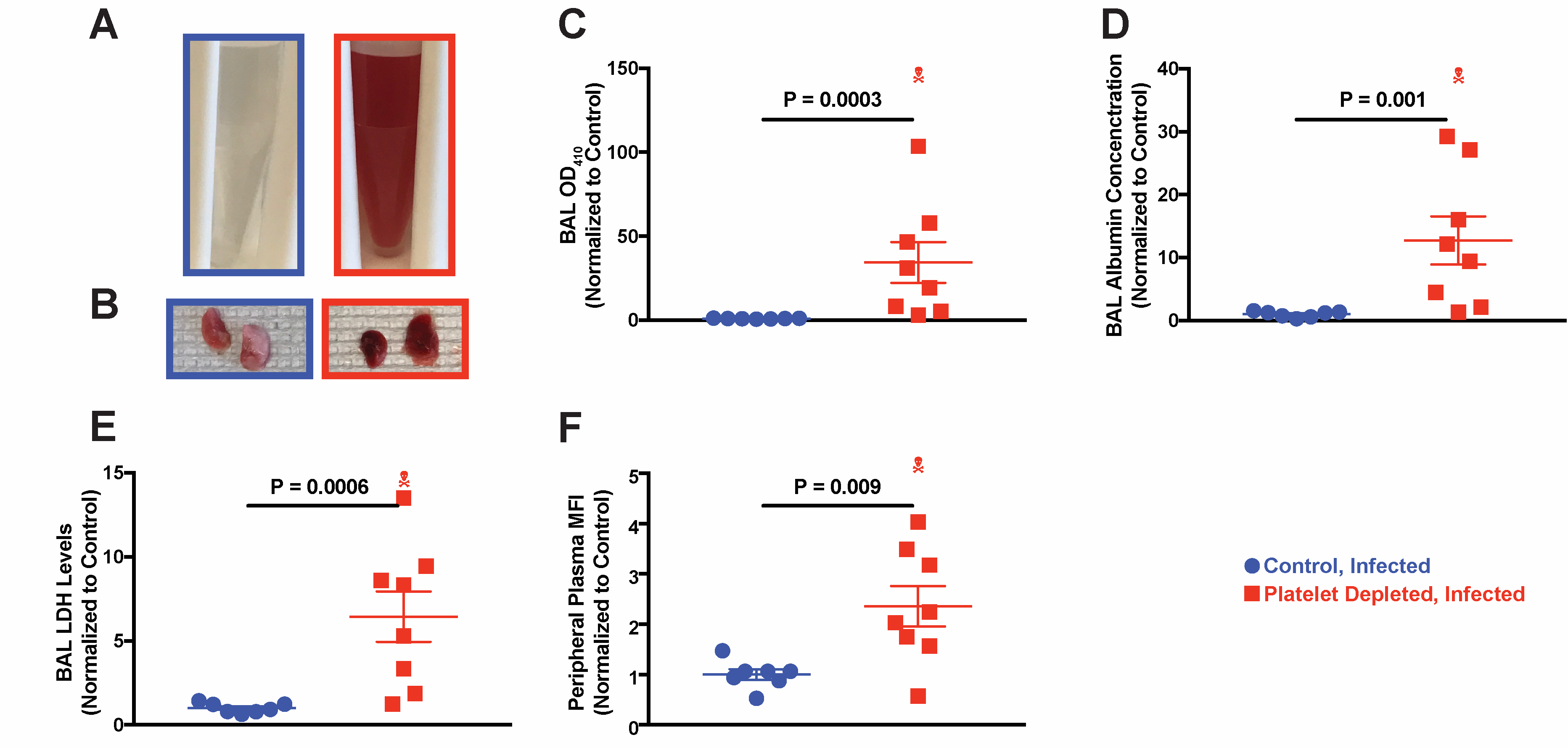

Supplement: S11 Fig — (A) Representative images of bronchoalveolar lavage fluid (BALF) and (B) perfused lungs 1 day after infection of Cre negative (blue) or iDTRPf4 (red) mice injected with DT and infected with ~3–6 x 10^7^ A. fumigatus CEA10 conidia. (C) Lung airway bleeding (from A) quantified by measuring BALF OD410 absorption, (D) airway vascular leakage determined by BALF albumin levels, (E) airway LDH levels, and (F) vascular permeability assessed by monitoring peripheral plasma fluorescence after intranasal installation of FITC-Dextran prior to euthanasia in thrombocytopenic and control mice. All data are expressed as relative values, with control mice (blue) having a value of 1.00, and thrombocytopenic mice (red) as a percentage of controls at approximately 24 hours post infection. Error bars are expressed with mean ± SEM. The skull symbol indicates a mouse died prior to harvest (excluded from analysis). Data from (C-F) are pooled from 2 experiments (n = 7–9 total mice per group). (TIF) [file ppat.1008544.s011.tif]

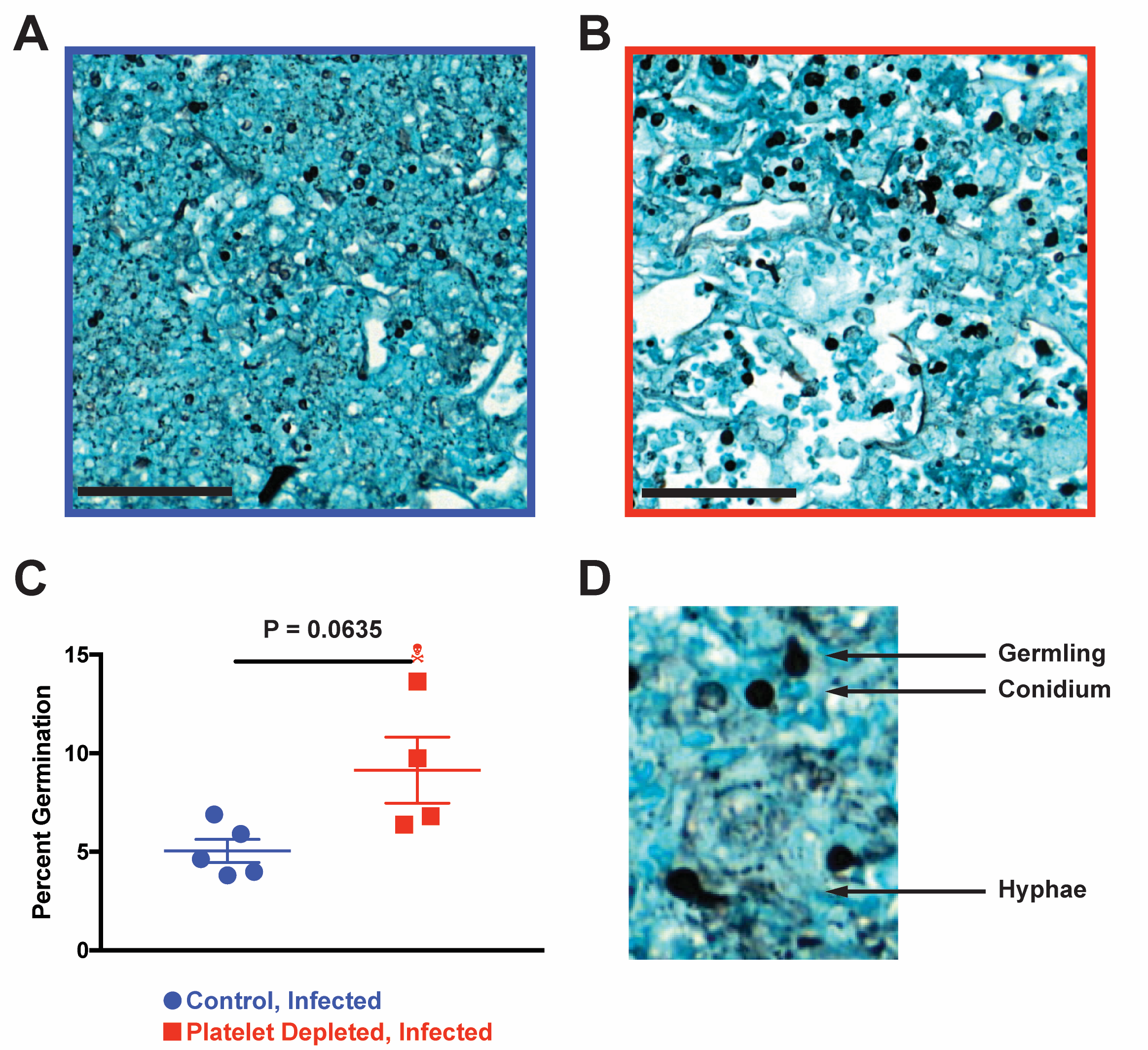

Supplement: S12 Fig — (A-B) Representative GMS staining of control infected (blue, A), or thrombocytopenic infected (red, B), C56BL/6J mouse lung 2 days after infection with ~3–6 x 10^7^ A. fumigatus CEA10 conidia in the AS model. The scale bars represent 50 μm. (C) Quantification of the germination rate in control infected (blue) and thrombocytopenic infected (red) C57BL/6J mice. Error bars are expressed with mean ± SEM. The skull symbol indicates a mouse died prior to harvest and was excluded from analysis. Data are from one experiment (n = 5 mice per group). (D) Germination rate in C was determined by counting all fungal particles and by dividing the number of germlings and hyphae by the total number. (TIF) [file ppat.1008544.s012.tif]
